# DNA hypermethylation modification promotes the development of hepatocellular carcinoma by depressing the tumor suppressor gene *ZNF334*

**DOI:** 10.1038/s41419-022-04895-6

**Published:** 2022-05-09

**Authors:** Dapeng Sun, Xiaojie Gan, Lei Liu, Yuan Yang, Dongyang Ding, Wen Li, Junyao Jiang, Wenbin Ding, Linghao Zhao, Guojun Hou, Jian Yu, Jie Wang, Fu Yang, Shengxian Yuan, Weiping Zhou

**Affiliations:** 1 The Third Department of Hepatic Surgery, Eastern Hepatobiliary Surgery Hospital, Naval Medical University, 225 Changhai Road Shanghai, Shanghai, 200438 China; 2 Center for Health Research, Guangzhou Institutes of Biomedicine and Health, Chinese Academy of Sciences, 190 Kaiyuan Avenue, Guangzhou, 510530 China; 3 The department of Medical Genetics, Naval Medical University, Shanghai, 200438 China

**Keywords:** Liver cancer, Mechanisms of disease

## Abstract

DNA methylation plays a pivotal role in the development and progression of tumors. However, studies focused on the dynamic changes of DNA methylation in the development of hepatocellular carcinoma (HCC) are rare. To systematically illustrate the dynamic DNA methylation alternation from premalignant to early-stage liver cancer with the same genetic background, this study enrolled 5 HBV-related patients preceded with liver cirrhosis, pathologically identified as early-stage HCC with dysplastic nodules. Liver fibrosis tissues, dysplastic nodules and early HCC tissues from these patients were used to measure DNA methylation. Here, we report significant differences in the DNA methylation spectrum among the three types of tissues. In the early stage of HCC, DNA hypermethylation of tumor suppressor genes is predominant. Additionally, DNA hypermethylation in the early stage of HCC changes the binding ability of transcription factor TP53 to the promoter of tumor suppressor gene *ZNF334*, and inhibits the expression of *ZNF334* at the transcription level. Furthermore, through a series of in vivo and in vitro experiments, we have clarified the exacerbation effect of tumor suppressor gene *ZNF334* deletion in the occurrence of HCC. Combined with clinical data, we found that the overall survival and relapse-free survival of patients with high *ZNF334* expression are significantly longer. Thus, we partly elucidated a sequential alternation of DNA methylation modification during the occurrence of HCC, and clarified the biological function and regulatory mechanism of the tumor suppressor gene *ZNF334*, which is regulated by related DNA methylation sites. Our study provides a new target and clinical evidence for the early diagnosis and sheds light on the precise treatment of liver cancer.

## Introduction

Primary liver cancer (PLC) is the fourth most prevalent malignant tumor in Chinese population with rapid development, low surgical resection rate, early recurrence potential, high metastatic ability, and high fatality rate [1–3]. Chronic inflammation caused by alcohol, hepatitis virus, metabolic disorders, etc. is generally considered to be the initiating factor for the occurrence of hepatocellular carcinoma (HCC) [4–7]. The inflammatory-cancer transformation exhibits as a chronic fibrosis process, often proceeding gradually from the liver dysplastic nodule (Dn) to malignance [8, 9]. In some well-differentiated PLC, the tumor tissue is surrounded by high-grade Dn to form a “nod in the nodule” structure [10]. Therefore, the liver Dn tissues are generally recognized as precancerous lesions that share more malignant features with liver cancer than cirrhotic tissues [11, 12]. However, few previous studies focused on the molecular changes in liver Dn, which is an important premalignant period of HCC [11, 13]. Early liver cancer may have strong metastatic characteristics, which exhibits as a high recurrence rate after radical resection of small liver cancer, indicating that early-stage liver cancer is accompanied by the acquisition of metastatic potential [14, 15]. From this aspect, research on precancerous lesions of liver cancer would help to find the driving events or molecules of the HCC, which is a significant premise to achieve the early diagnosis and surgical intervention of liver cancer.

Epigenetic modifications, including histone modification, DNA methylation modification, chromatin remodeling, and non-coding RNA regulation, are crucial regulatory mechanisms for cells to adapt to changes in the external environment [16, 17]. Studies have shown that DNA methylation modification disorder is a common feature in PLC [18, 19]. Genome-wide hypomethylation in HCC causes genome instability, which in turn leads to mutations or expression alterations of tumor-related genes [20]. In the meanwhile, abnormal methylation alternation of tumor-related genes may lead to the inhibition of tumor suppressor genes (TSGs) or the promotion of the oncogenes, thereby contributing to the occurrence and development of liver cancer [21]. Previous studies have also shown that during the formation process of hepatitis B virus-related liver cancer, the abnormality of key DNA methylation enzymes such as DNA methyltransferases (DNMTs) leads to the inactivation of TSGs and the activation of oncogenes, accelerating the development of liver cancer [22, 23]. DNA methylation modification may exhibit different patterns in different stages of liver cancer. In the comparison between early and advanced liver cancer, the lineage of DNA methylation is the most extensively different, which also indicates the abnormalities of the methylation modification in HCC are secondary changes after the occurrence of liver cancer [24].

Previous studies usually ignored the individual heterogeneity, preferring the same type of tissue from different patients for research [18, 25]. Although the tissue types are the same, the genetic background is far from similar. In the sequential process from hepatitis to cirrhosis to cancer, the continuous changes in the genome are easily ignored because of the discontinuity of different types of tissues. Therefore, we recruited 5 patients who were pathologically diagnosed as early-stage liver cancer with dysplastic nodules. The liver fibrosis tissues, Dn tissues and HCC tissues of each patient were taken as a group and detected by methylation chip. Since the genetic backgrounds of samples are the same, the heterogeneity within the group is controlled to a minimum, which can better clarify the sequential changes of epigenetic modifications during the “inflammation-to-cancer transformation” process.

Overall, our study partly reveals the dynamic changes of DNA methylation from precancerous to the early stages of liver cancer, finds potential driver DNA methylation sites in liver cancer, and clarifies the biological functions and regulatory mechanisms of downstream genes regulated by related DNA methylation sites. Collectively, these results provide a new target and clinical evidence for early diagnosis and precise treatment of liver cancer.

## Results

### Hypermethylation of TSGs occurs in the early stage of liver tumorigenesis

**Fig. 1:**
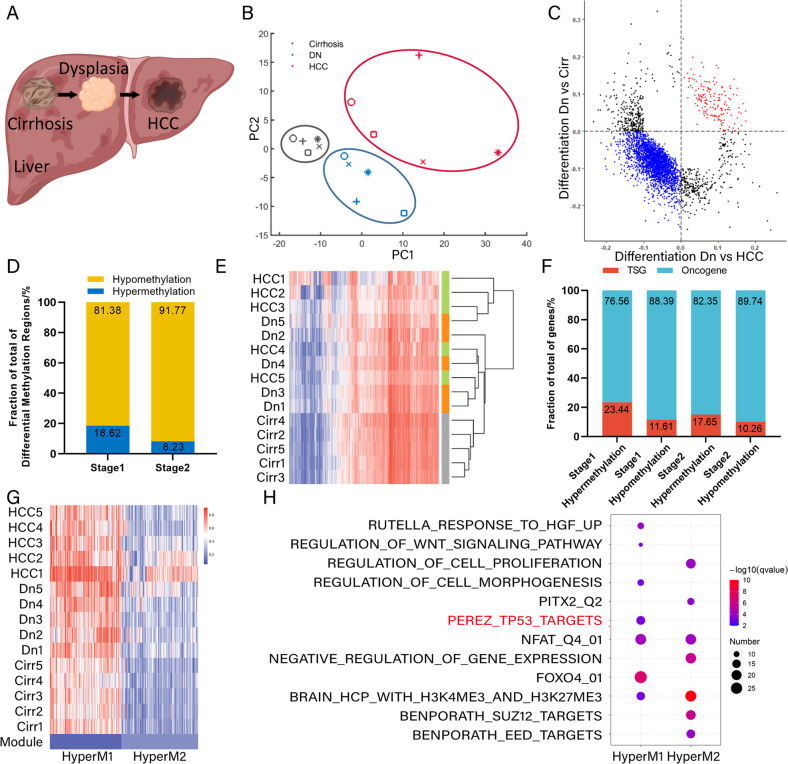
DNA methylation analysis reveals epigenetic changes in the early stage of liver tumorigenesis. **A** A diagram of cirrhosis, dysplastic nodules and HCC from each patient. **B** Principal component analysis (PCA) shows different methylation spectrum of cirrhosis, dysplastic nodules and HCC tissues. PC1 and PC2 indicate principal component 1 and 2, respectively. **C** Comparison of differentially methylated regions in the first stage from cirrhosis to dysplastic nodules and the second stage from dysplastic nodules to HCC. **D** Fraction of differentially methylated regions in the first and second stages of liver tumorigenesis. **E** Clustering analysis of cirrhosis, dysplastic nodules and HCC based on differentially methylated sites. **F** Fraction of oncogenes and tumor suppressor genes (TSGs) in differentially methylated sites in the first and second stages. **G** K-means clustering of hypermethylated regions. **H** Gene function enrichment analysis of hypermethylated regions using GSEA software.

In this study, we enrolled five liver cancer patients. Specially, we obtained cirrhosis, liver dysplastic nodules and early HCC tissues from each patient and set them as a group (Fig. 1A). The histological types of tissues were confirmed by HE staining (Fig. S1G). Genomic DNA methylation status in these fifteen tissues was measured. By analyzing DNA methylation profiles, we obtained the methylation levels of all 23,638 CpG sites across the whole genome. Principal component analysis (PCA) indicates that cirrhosis, dysplastic nodules and HCC tissues manifest distinct methylation spectra (Fig. 1B). In the first stage from cirrhosis to dysplastic nodules (Stage 1) and the second stage from dysplastic nodules to HCC (Stage 2), we separately identified 6808 and 2956 differentially methylated sites (Fig. S1D, S1E). Among differentially methylated sites, 3762 (55.25%) and 635 (21.48%) were hypermethylated in Stage 1 and Stage 2, respectively (Fig. S1C, S1D). Additionally, we further identified 108 (18.62%) hypermethylated and 472 (81.38%) hypomethylated regions in Stage 1 (Figs. 1C, D, S1A, S1B). In Stage 2, there were 26 (8.23%) hypermethylated and 290 (91.77%) hypomethylated regions (Fig. 1D). Most of these differentially methylated regions were situated at the exonic, intronic and intergenic regions (Fig. S1F). Based on all differentially methylated regions, clustering analysis shows that cirrhosis samples are set apart from dysplastic nodules and HCC, and dysplastic nodules are more similar to HCC in this aspect (Fig. 1E). Next, we identified the genes associated with differentially methylated regions. In Stage1, 76.56% hypermethylated genes were oncogenes, and the proportion in Stage 2 was 82.35% (Fig. 1F). A higher fraction of TSGs was hypermethylated rather than hypomethylated in both Stage1 and Stage 2 (Fig. 1F). By K-means clustering, the differential hypermethylated regions were separated into two groups according to their methylation levels (Fig. 1G). Function enrichment analysis indicates that the genes with higher methylation levels are significantly regulated by TP53 transcription factors and related to FOXO4 (Fig. 1H). We also observed that WNT signaling pathway and cell proliferation were regulated through hypermethylation during HCC progression. Additional enrichment analysis confirmed that these functions were enriched in hypermethylated genes (Supplementary Table 1). After looking up into single cell RNA sequencing (scRNA-seq) data in the Human Protein Atlas database (HPA, http://proteinatlas.org), we found that the TP53 (HPA, ENSG00000141510) was abundantly while FOXO4 (HPA, ENSG00000184481) was barely expressed in hepatocytes and cholangiocytes (Fig. S2). It is acknowledged that these hepatic parenchymal cells are the key participants in liver cancer formation process, and TP53 protein takes part in various process in tumorigenesis, whereas the tiny expression of FOXO4 may have limited influence on the liver tumorigenesis. Hence, we mainly focused on TP53 protein in the following study.

### Hypermethylation of *ZNF334* promoter maintains throughout the process of inflammatory-to-cancer transformation in DEN-induced carcinogenesis mice model

**Fig. 2:**
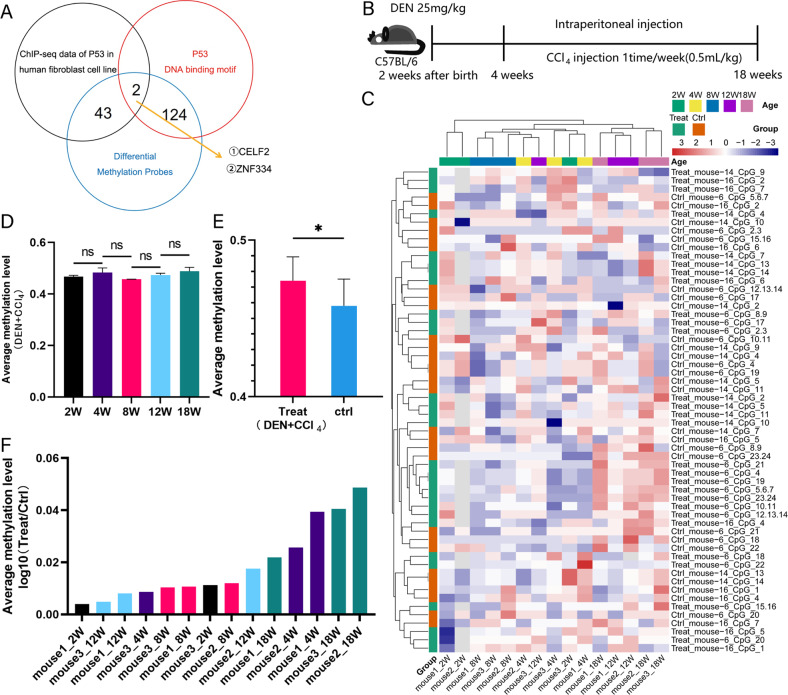
DEN-induced carcinogenesis mice model reveals hypermethylation of *ZNF334* promoter. **A** Venn diagram of the TP53 target genes in three databases. **B** Schematic diagram of how DEN-induced carcinogenesis mice model was established. **C** Heat map of methylation level of *Zfp334* promoter in treatment and control group at indicated time. The methylation level was detected through three PCR reactions (mouse-6, mouse-14, and mouse-16), the first reaction includes 24 CpG islands (mouse-6_CpG_1-24), second includes 14 CpG islands (mouse-14_CpG_1-14), and the last includes 7 CpG islands (mouse-16_CpG_1-7). **D** The average methylation degree of *Zfp334* promoter in treatment group at indicated time. **E** The average methylation degree of *Zfp334* promoter in treatment group is higher than that of control group. **F** The ratio of methylation degree of *Zfp334* promoter in treatment and control group (Treat/ctrl) at indicated time. *p < 0.05.

Next, the differentially methylated sites of the EPIC chip were integrated with the TP53 protein ChIP-seq data (GSE55727) and TP53 DNA binding motif data from JASPAR database. The results showed that *CELF2* and *ZNF334* were the TP53 target genes shared by the three data (Fig. 2A). Analysis showed that *CELF2* expression had no correlation with HCC patients’ prognosis (data not shown). Yet, the LIHC cohort in the TCGA database exhibited a correlation between *ZNF334* expression and patients’ prognosis (Fig. S1F). To verify whether *ZNF334* promoter was hypermethylated during the inflammatory-to-cancer transformation process, we constructed a DEN-induced carcinogenesis mice model (Fig. 2B) and examined the DNA methylation level of *ZNF334* (also called *Zfp334* in mice) promoter at different time point after CCl4 injection (2w, 4w, 8w, 12w, and 18w after first CCl4 injection, respectively). A heatmap was used to illustrate the methylation status of *Zfp334* promoter region with different PCR reaction. However, due to the slight change in the methylation level, there was no clear-cut pattern of methylation (Fig. 2C). The slight change in the methylation level of *Zfp334* promoter region at different time point was further confirmed (Fig. 2D). While when we compared the methylation level of the treated group and control group, the results showed that the *Zfp334* promoter of the treatment group had higher DNA hypermethylation level than the control group did (Figs. 2E, F, S3A–C), indicating that hypermethylation of *Zfp334* promoter is established at the very beginning of tumorigenesis and remains at a high level all through the process of inflammatory-to-cancer transformation in mice liver cancer model.

### Hypermethylation of *ZNF334* promoter inhibits the expression of ZNF334 in HCC patients

**Fig. 3:**
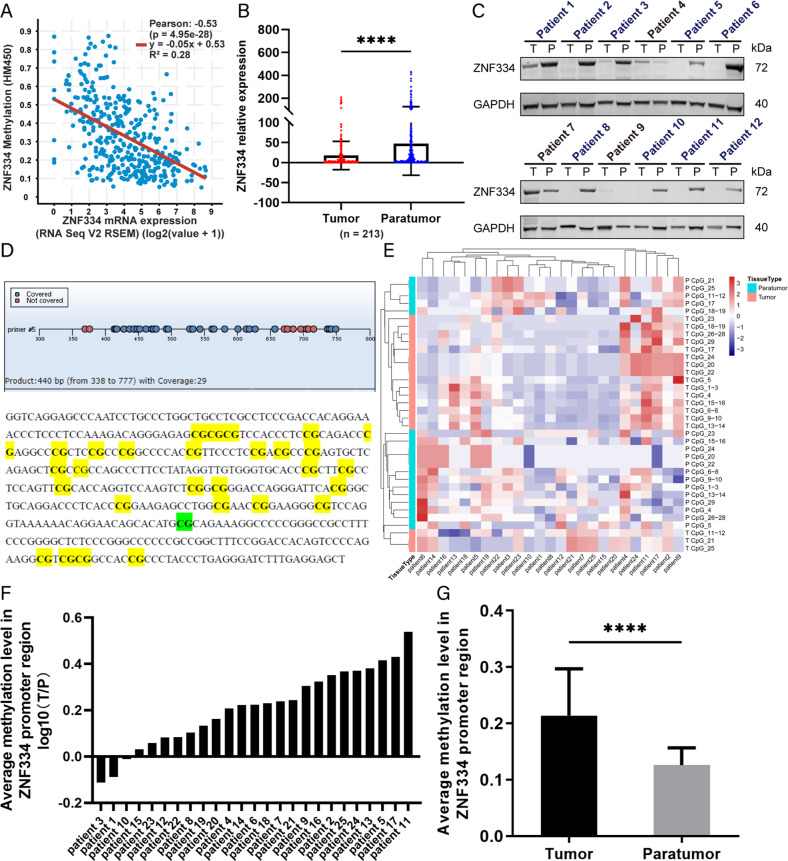
Promoter hypermethylation and expression of ZNF334 in HCC patients. **A** A negative correlation between *ZNF334* methylation level and its mRNA expression (Pearson *r* = −0.53, *R*^2^ = 0.28) **B** mRNA expression of *ZNF334* in cancer and adjacent tissues of 213 liver cancer patients. **C** Western-blot diagram of ZNF334 expression in cancer and adjacent tissues of 12 liver cancer patients. **D** Methylation mass spectrum primers of *ZNF334* promoter region and mass spectrometry detection product sequence (green indicates the EPIC chip probe cg07139762 site, which is in the *ZNF334* promoter region; yellow indicates the MassARRAY methylation mass spectrometry detection sites, a total of 29). **E** Heat map of methylation level of *ZNF334* promoter region in cancer and adjacent tissues of 25 patients with liver cancer. **F** The ratio of methylation degree of *ZNF334* promoter in cancer and adjacent tissues (T/P) of 25 patients with liver cancer. **G** The average methylation degree of *ZNF334* promoter in cancer and adjacent tissues of 25 patients with liver cancer. ****p < 0.0001.

Firstly, we found a negative correlation between the *ZNF334* methylation level and *ZNF334* mRNA expression (Pearson *r* = −0.53, *p* = 4.95e-28; TCGA, Firehose Legacy, 373 samples) (Fig. 3A). For tissues from patients, the expression of *ZNF334* at mRNA level in the tumor tissues significantly decreased by detecting the cancer and adjacent tissues of 213 liver cancer patients (Fig. 3B). Twelve patients’ samples were used to examine the *ZNF334* protein level, which showed a descending trend in tumor tissues compared to the paratumor tissues (Fig. 3C). In addition, MassARRAY methylation detection primers (Fig. 3D) for the human *ZNF334* promoter region (the 2000bp region upstream of the transcription start site, TSS) were designed and applied to the detection of 25 paired tissues out of the 213-patients cohort in our hospital. The results manifested that the methylation degree of *ZNF334* promoter region in cancer tissues was significantly higher than that in adjacent tissues of the same patient (Fig. 3E–G).

### Transcription factor TP53 regulates the expression of TSG *ZNF334*

**Fig. 4:**
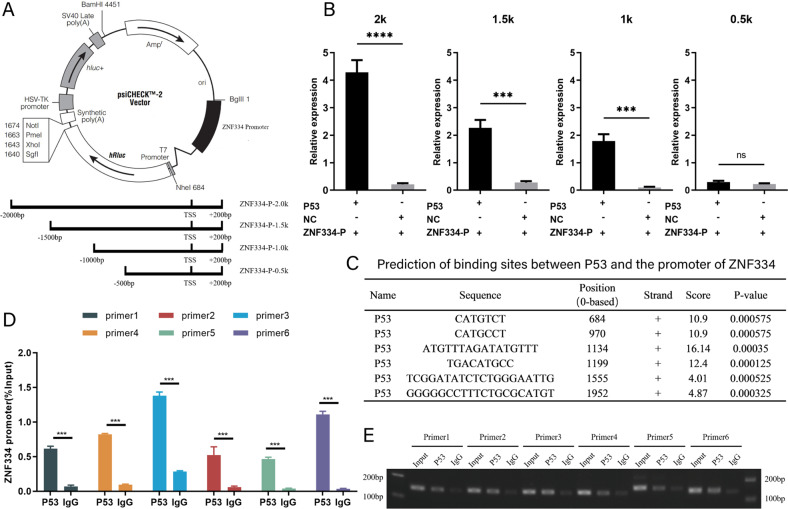
Transcription factor TP53 regulates the expression of TSG ZNF334. **A** Diagram of the construction of dual luciferase plasmid (upper) and diagram of truncation of the promoter region (lower). **B** Fluorescence intensity expressed by dual luciferin of plasmids with different lengths of *ZNF334* promoter region. **C** Prediction of binding sites between TP53 and *ZNF334* promoter region (TP53 binding site sequence GGGGGCCTTTCTGCGCATGT contains the EPIC chip probe cg07139762). **D** Conditions of TP53 binding to different *ZNF334* promoter sites. **E** Electrophoresis diagram of PCR product by chromatin immunoprecipitation experiment. ***p < 0.001; ****p < 0.0001.

To further investigate the relationship between TP53 and *ZNF334*, we constructed a dual luciferase reporter plasmid system, which contained the full length of the promoter region of *ZNF334* (2000bp DNA fragment, 1500-2000bp upstream of the TSS, also containing the EPIC chip probe cg07139762) (Fig. 4A upper). The results showed that, compared with the control plasmid, the plasmids co-transfected with TP53 and *ZNF334* promoter could significantly increase the expression abundance of luciferase in HepG2 cell line, which demonstrated that TP53 could bind to the promoter region of *ZNF334* and promote transcription. The promoter region of *ZNF334* was further truncated (Fig. 4A lower) for dual luciferase reporter gene experiments. The results showed that, TP53 protein could still bind to the truncated 1500bp- or 1000bp-promoter and induce their expression; however, the luciferase did not express when *ZNF334* promoter region was truncated to 500 bp length (Fig. 4B). Therefore, we speculated that there might be multiple binding sites between TP53 and *ZNF334* promoter ranging from 500 to 2000 bp upstream of the TSS. In order to further verify whether the TP53 bound to the *ZNF334* promoter, we used JASPAR (transcription factor binding site prediction website) to predict the binding sites between TP53 and *ZNF334* promoter (Fig. 4C). Interestingly, the EPIC chip probe cg07139762 was also located in the TP53 binding site sequence (GGGGGCCTTTCTGCGCATGT). Subsequently, primers were designed for the predicted sites to perform chromatin immunoprecipitation (ChIP) experiments. The results of ChIP experiments showed that in Huh7 cell, the binding of TP53 to the *ZNF334* promoter region was significantly higher than that of IgG, which proved that TP53 could bind to the *ZNF334* promoter region (Fig. 4D, E).

### *ZNF334* promotes liver cancer cell apoptosis, inhibits the proliferation and tumor formation ability of liver cancer cells in vitro

**Fig. 5:**
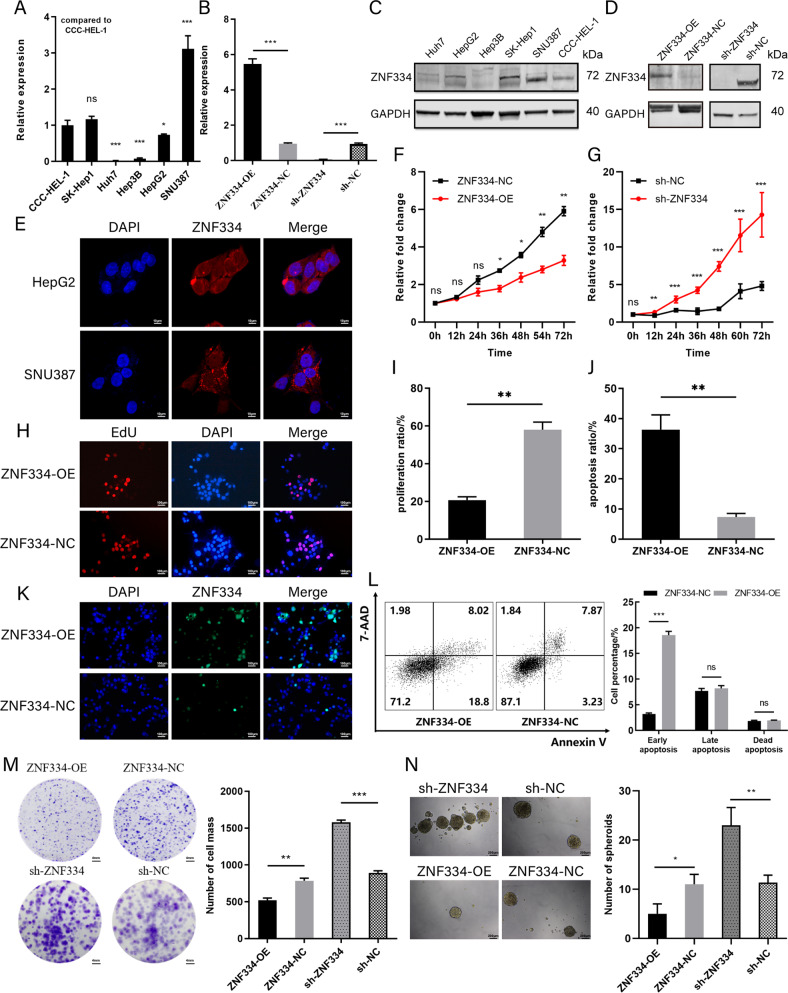
ZNF334 promotes cell apoptosis, inhibits proliferation and stemness of liver cancer cells in vitro. **A** mRNA expression of *ZNF334* in different cell lines. **B** mRNA expression of *ZNF334* in overexpression Huh7 cell line (ZNF334-OE) and SNU387 interfering cell line(sh-ZNF334). **C** Protein expression of ZNF334 in different cell lines. **D** Protein expression of ZNF334 in overexpression Huh7 cell line (ZNF334-OE) and SNU387 interfering cell line(sh-ZNF334). **E** Representative images of ZNF334 expression in HepG2, SNU387 (immunofluorescence staining, ×200, blue fluorescence represents nucleus, red fluorescence represents ZNF334). **F** Cell proliferation curve of ZNF334-OE and ZNF334-NC by CCK8 in Huh7 cell line. **G** Cell proliferation curve of sh-ZNF334 and sh-NC by CCK8 in SNU387 cell line. **H** Proliferation ratio of ZNF334-OE and ZNF334-NC by EdU proliferation assay. **I** Apoptosis ratio of ZNF334-OE and ZNF334-NC by TUNEL apoptosis assay. **J** Representative images of proliferation of ZNF334-OE and ZNF334-NC by EdU (immunofluorescence staining, ×200, blue fluorescence represents nucleus, red fluorescence represents proliferating cells). **K** Cell apoptosis of ZNF334-OE and ZNF334-NC by flow cytometry. **L** Representative images of apoptosis of ZNF334-OE and ZNF334-NC by EdU (immunofluorescence staining, ×200, blue fluorescence represents nucleus, green fluorescence represents nucleus undergoing apoptosis). **M** Representative images and statistical analysis of ZNF334-OE, ZNF334-NC, sh-ZNF334, and sh-NC by plate clone formation assay. **N** Representative images and statistical analysis of ZNF334-OE, ZNF334-NC, sh-ZNF334, and sh-NC by sphere formation assay. ns, not significant; *p < 0.05; **p < 0.01; ***p < 0.001.

**Fig. 6:**
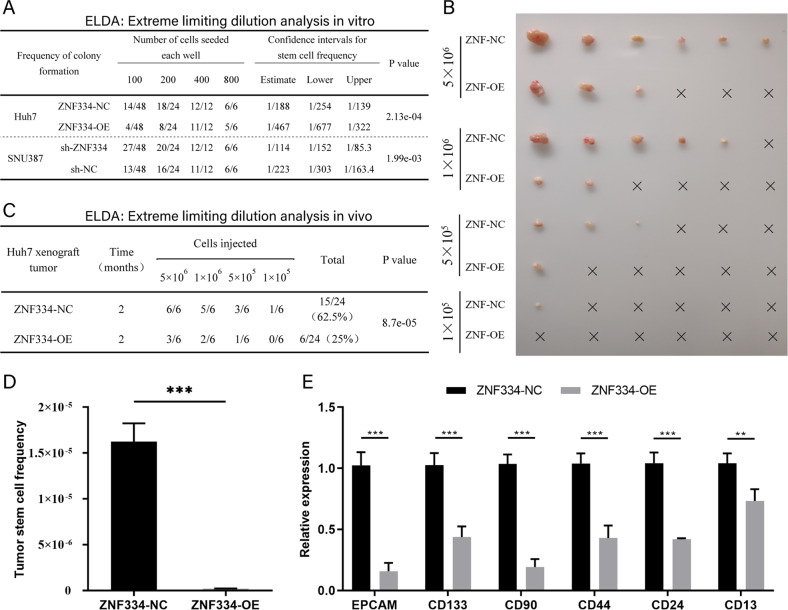
ZNF334 inhibits tumor formation in vivo. **A** Extreme limiting dilution analysis of ZNF334-OE, ZNF334-NC, sh-ZNF334, and sh-NC in vitro. **B** The number and size of subcutaneous tumors in nude mice with varying number of ZNF334-OE and ZNF334-NC injection. **C** Extreme limiting dilution analysis in vivo. **D** Proportion of tumor stem cells calculated by ELDA software in subcutaneous tumor tissues of nude mice with ZNF334-OE and ZNF334-NC cell injected. **E** The mRNA expression of different stem molecules of tumor stem cells in subcutaneous tumor tissues of nude mice with ZNF334-OE and ZNF334-NC cell injected by realtime qPCR. **p < 0.01; ***p < 0.001.

To further clarify the biological feature of *ZNF334* in HCC, the expression of *ZNF334* in different HCC cell lines (Huh7, HepG2, Hep3B, SK-Hep1, SNU387) and a normal liver cell line (CCC-HEL-1) were measured (Fig. 5A, C). The results of RT-qPCR and western blot both showed that *ZNF334* expressed lower in Huh7 and Hep3B, and higher in SK-Hep1, SNU387, HepG2, and CCC-HEL-1 (Fig. 5A, C). Therefore, based on the expression level of *ZNF334* in different cells, we used lentiviral vectors to construct a stable transgenic *ZNF334* overexpressing Huh7 cell line (ZNF334-OE) and its control cell line (ZNF334-NC) (Fig. 5B, D); a cell line of which *ZNF334* was stably knocked down (sh-ZNF334) and its control cell line (sh-NC) were constructed using SNU387 cell line (Fig. 5B, D). The results of immunofluorescence showed that ZNF334 was expressed variously in the nucleus and cytoplasm in HepG2 and SNU387 cell lines (Fig. 5E). The cell proliferation assay (CCK8) showed that compared with their negative control cells, the proliferation ability of ZNF334-OE was significantly reduced (Fig. 5F), while improved in sh-ZNF334 (Fig. 5G). Similarly, the proliferation rate of ZNF334-OE marked with EdU was much lower than that of the control group in the case of seeding the same number of cells (Fig. 5H, I). Besides, we also found that the proportion of ZNF334-OE cells undergoing apoptosis increased significantly when the same number of cells were inoculated (Fig. 5J, K). And compared with the control group, the elevated apoptosis of ZNF334-OE cells was mainly attributed to the increase of early apoptosis cells (Fig. 5L). In addition, plate clone formation assay also showed that compared with ZNF334-NC, fewer tumor clusters with smaller size were formed in ZNF334-OE, while the clone formation ability of sh-ZNF334 was significantly higher than that of sh-NC, which further proved that overexpression of *ZNF334* could inhibit the proliferation of tumor cells (Fig. 5M). We further evaluated the effects of different expression levels of *ZNF334* on the tumor formation ability of HCC cell lines. The results of the sphere formation assay showed that the number of cell clumps formed by ZNF334-OE decreased, while the spheronization ability of sh-ZNF334 cells was significantly higher than that of sh-NC (Figs. 5N, S4). Limiting dilution assay (LDA) in vitro showed that the proportion of ZNF334-OE tumor stem cells was significantly lower than that of the control group, while the number of cell clusters formed by sh-ZNF334 was significantly higher than that of the control group, further indicating that overexpression of *ZNF334* could reduce the tumor formation ability of tumor cells (Fig. 6A).

### *ZNF334* inhibits tumor formation in vivo

Limiting dilution assay in vivo showed that, under the condition of injecting the same number of tumor cells, the tumors formed under the mice skin with ZNF334-NC was more than that of the ZNF334-OE group, and a certain number of tumors could still be formed at a low cell concentration injected (5 × 10^5^) (Fig. 6B). On the other hand, fewer subcutaneous tumors were formed in the mice with ZNF334-OE injected, and even no tumor formed at 1 × 10^5^ concentration (Fig. 6B). Using Extreme Limiting Dilution Analysis (ELDA, a website analysis tool), we found that the proportion of stem cells in ZNF334-OE cells was significantly lower than that of the control group (Fig. 6C, D). Further measuring the markers related to the tumor formation ability in mice subcutaneous tumors, we found that EpCAM, CD13, CD24, CD44, CD90 and CD133 in ZNF334-OE cells was significantly lower than that of the control group, which was consistent to the phenotype of the mice model (Fig. 6E).

### The overall survival and relapse-free survival of patients with higher expression of *ZNF334* are longer

**Fig. 7:**
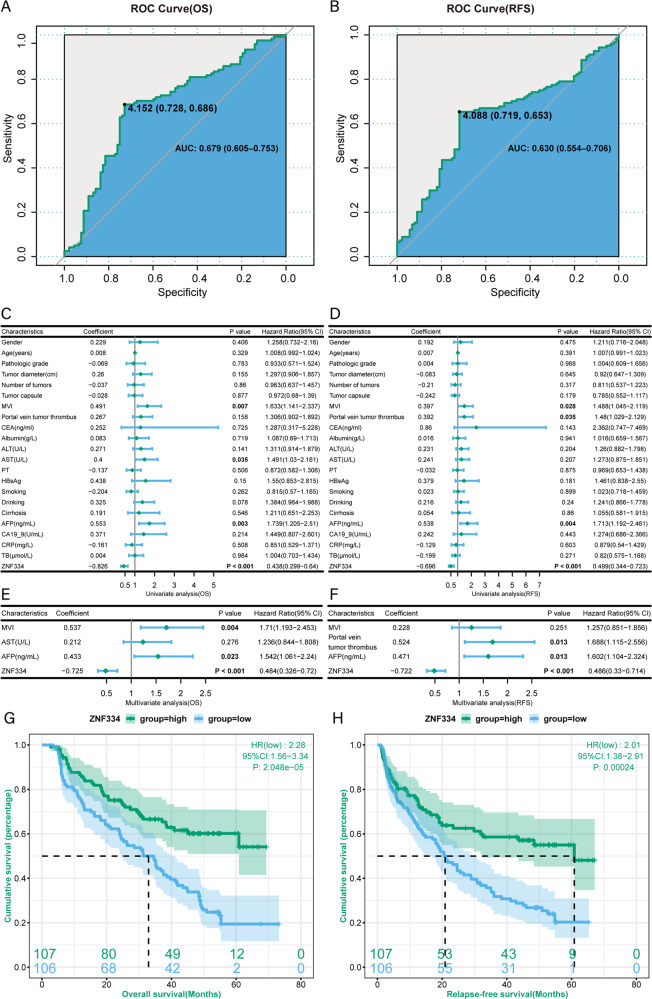
The prognosis of patients with higher expression of ZNF334 are better. **A** Receiver operating characteristics curve showing the *ZNF334* cut-off value associated with overall survival (OS). **B** Receiver operating characteristics curve showing the *ZNF334* cut-off value associated with relapse-free survival (RFS). **C** Univariate analysis of factors associated with overall survival of 213 HCC patients. **D** Univariate analysis of factors associated with relapse-free survival of 213 HCC patients. **E** Multivariate analysis of factors associated with overall survival of 213 HCC patients. **F** Multivariate analysis of factors associated with relapse-free survival of 213 HCC patients. **G** Kaplan–Meier analysis showing the overall survival of HCC patients with diverse *ZNF334* expression. **H** Kaplan–Meier analysis showing the relapse-free survival of HCC patients with diverse *ZNF334* expression.

In order to further investigate the relationship between *ZNF334* and the prognosis of liver cancer patients, we collected the tumor tissues from 213 liver cancer patients and detected the expression of *ZNF334* at the mRNA level (Fig. 4B). According to Youden index (OS_Youden index_ = 4.152, RFS_Youden index_ = 4.088, Fig. 7A, B), patients were divided into *ZNF334* high expression group (*n* = 107) and low expression group (*n* = 106). Chi-square showed that *ZNF334* was related to tumor growth-related indicators-alpha-fetoprotein (AFP), number of tumors and tumor capsule, and was also concerned with metastasis-related indicators-portal vein tumor thrombus. Compared with the *ZNF334* high expression group, the proportion of people with high serum AFP( > 400 ng/ml), tumor number and portal vein tumor thrombus was greater, while the proportion of people with tumor capsule was lower (Supplementary Table 3). These results suggested that the *ZNF334* level was negatively correlated with the malignancy of liver cancer.

Cox univariate analysis found that *ZNF334*, AFP, AST, and MVI were related to overall survival (OS) in patients with liver cancer (Fig. 7C); *ZNF334*, AFP, MVI, and portal vein tumor thrombus were related to postoperative relapse-free survival (RFS) (Fig. 7D). Cox multivariate analysis found that AFP > 400 ng/ml, low *ZNF334* expression and MVI were independent risk factors that affected the patients’ OS (Fig. 7E); while AFP > 400 ng/ml, low *ZNF334* expression and portal vein tumor thrombus were the independent risk factors that affected patients’ RFS (Fig. 7F). OS and RFS Kaplan-Meier Curve showed that the OS and RFS of the *ZNF334* high expression group were higher than those of the low expression group (*p*_OS_ < 0.001; *p*_RFS_ < 0.001) (Fig. 7G, H).

## Discussion

Previous studies have shown that changes in genomic epigenetics are closely related to the occurrence of cancer [26–28]. Here, we used the EPIC methylation chip to systematically detect the DNA methylation conditions in fibrosis tissues, Dn tissues and early hepatocellular carcinoma tissues of the same patient. The results showed that the DNA methylation lineages in the three tissues were significantly different. In the early stage of liver tumorigenesis (Stage 1), the proportion of DNA hypermethylation was higher than that of hypomethylation, and the relevant sites were mainly located in TSGs, which suggests that the hypermethylation of TSGs may be the main epigenetic feature in the early stage of liver tumorigenesis process, promoting the occurrence of liver cancer to a certain extent. Previous studies have reported that in tumor tissues, the increased hypermethylation of TSGs and the decreased genomic methylation status are primary drivers of cancer [29–33], which is consistent with our results. Here, we found that the methylation level of the whole genome differed in the three kinds of liver tissues, yet, according to the morphophenotypic changes, the dysplastic nodules could also be divided into low-grade and high-grade dysplastic nodules, the methylation level of which might have slight differences from each other [13]. In our study, we did not distinguish these two kinds of tissues because of the scarcity and low detection rate of the dysplastic nodule samples, and we admitted that it was one of the limitations of our work. It remained to be further studied with a larger sample enrollment in the future.

As an important transcription factor in the body, TP53 participates in regulating cell growth, maintaining genome stability, and inhibiting tumor angiogenesis [34]. As a tumor suppressor gene, wild-type *TP53* can inhibit the occurrence of tumors [35], while mutant *TP53* affects the regulation level of downstream molecules and promotes tumorigenesis. Studies have shown that mutant *TP53* exists in over 50% of tumor tissues [36], contributing to the occurrence and development of tumors [37]. In this study, k-means clustering analysis and gene function enrichment analysis were performed in the DNA hypermethylated regions in Stage 1, and it was found that the target gene of TP53 was significantly enriched in the DNA hypermethylation segment.

Additionally, studies have shown that the DNA methylation modifications can affect the transcription of TSGs [17, 25, 32]. Variations in DNA methylation modification status will cause changes in the binding of the key transcription factors to downstream TSGs [38]. The hypermethylation of TSGs’ promoter dampens the binding capacity of transcription factor, and leads to the gene silence with a decrease in transcription [39–42], which is recognized as an mechanism of TSGs losing their biological functions. In this study, we compared the differential methylated site data with the relevant data in the existing database, and screened out the target gene *ZNF334* which was the downstream gene of TP53. To further verify the regulation mechanism of the transcription factor TP53 on the gene *ZNF334*, we performed the dual luciferase reporter gene experiment and ChIP experiment to prove that the TP53 can regulate the expression of *ZNF334* by binding to the *ZNF334* promoter. And meanwhile, in clinical samples, we used MassArray Sequenom detection to further verify the DNA methylation changes of *ZNF334* promoter in cancerous tissues and adjacent tissues. The results revealed that in tumor tissues, the extent of hypermethylation modification in the *ZNF334* promoter increased, which may be the main reason for the declined binding of TP53.

In order to further explore the biological role of *ZNF334* in the development of liver cancer, a series of in vivo and in vitro experiments were designed to explore the function of *ZNF334*. The results showed that *ZNF334* could significantly promote the apoptosis of liver cancer cells, inhibit the proliferation and stemness of liver cancer cells in vitro, and significantly inhibit the occurrence of tumors in the subcutaneous tumor-bearing mouse model. In addition, combined with clinical samples and follow-up data, we found that compared with paracancerous tissues, the expression of *ZNF334* in tumor tissues of liver cancer patients was significantly reduced. Patients with high *ZNF334* expression had longer recurrence-free survival and overall survival than those with low expression.

As we all know, *ZNF334* is a member of the zinc finger protein family and immunofluorescence results shows that *ZNF334* is expressed in both nucleus and cytoplasm. Hence, we deduce that *ZNF334* is likely to function as a transcription factor and bind to downstream genes’ promoters, thus affecting gene expression; it may also work as a secreted protein or intracellular protein to interact with other proteins in the cell and affect its function. Therefore, the molecular mechanism downstream of *ZNF334* needs further research and confirmation.

In conclusion, our study partly elucidated a sequential alternation of DNA methylation modification during the occurrence of HCC, provided some clues for revealing the temporal and spatial characteristics of DNA hypermethylation modification and firstly identified the regulatory mechanism of TP53 on its downstream target gene *ZNF334*. Furthermore, we explored the biological role of tumor suppressor gene *ZNF334* in the occurrence of liver cancer and provided a new target and clinical evidence for the early diagnosis and precise treatment of liver cancer.

## Methods

### Human liver tissue

A total of 213 specimens of patients with HCC from September 2012 to September 2019 were selected from the liver tissue specimen bank of Eastern Hepatobiliary Surgery Hospital, of which 5 cases were HBV-related HCC patients preceded with liver cirrhosis, and pathologically identified as early-stage with liver dysplastic nodules. The inclusion criteria for HCC patients are listed as followed: ①Patients were clinically diagnosed as liver cancer and the pathological diagnosis after liver resection was hepatocellular carcinoma;②Radiotherapy, neoadjuvant chemotherapy and other treatment measures were not taken before operation;③All patients underwent hepatectomy for liver cancer, and pathology afterwards confirmed R0 resection;④Age ≥18 years old and ECOG score≤2 points;⑤There was no distant metastasis;⑥The clinicopathological data were complete and the postoperative follow-up data were complete. HCC tumor tissues (the site where the tumor grows vigorously) and the matched adjacent tissues (more than 2 cm from the tumor margin) were collected. All samples were standardized immediately after operation and stored in liquid nitrogen for a long time. Part of the tissues were formalin fixed and paraffin embedded, sectioned for consecutive slices, and stained by hematoxylin-eosin staining. All patients who provided liver samples signed an informed consent form before the operation and reported to the hospital ethics committee for approval.

### Detection and analysis of DNA methylation

Liver tissues were formalin fixed and paraffin embedded, sectioned for consecutive slices, and stained by hematoxylin-eosin staining. For consecutive sections, the first and last section was HE stained and verified by at least two professional pathologists specialized in liver pathology. Then the genomic DNA of the in-between sections of liver fibrosis tissues, dysplastic nodule tissues, and early liver cancer tissues of 5 patients were extracted using QIAamp FFPE DNA Kit (QIAGEN), and then treated with sulfite to make unmethylated C into U, and the methylated C remains unchanged. DNA sample quality inspection and methylation microarray detection were carried out in accordance with the standard procedures of Infinium^®^ Methylation EPIC. R package ‘RnBeads’ was used to analyze microarray data and identify differentially methylated sites (absolute delta β > 0.1 and *p* < 0.01). Considering that there is a limited number of differentially methylated positions with delta β > 0.3, we chose the threshold of differentially methylated positions is more than 0.1. If delta β is positive, differentially methylated sites are defined as hypermethylated sites. Otherwise, differentially methylated sites are defined as hypomethylated sites. Principal component analysis, k-means clustering analysis, and heatmap of DNA methylation levels were performed through R platform. We calculated silhouette coefficient to determine the optimal number of clusters in k-means clustering analysis through R package ‘cluster’. Gene function enrichment analysis were conducted using GSEA (gene set enrichment analysis) and AutoCompare_ZE [43, 44]

### Cell culture and experimental conditions

Human HCC cell lines (Huh7, HepG2, Hep3B, Sk-Hep1, SNU387) and normal liver cell lines (CCC-HEL-1) were purchased from Chinese Academy of Sciences Cell Bank The above-mentioned cells were all recently authenticated by STR and without mycoplasma contamination and cultured in a high-sugar DMEM medium or MEM medium containing 10% FBS at 37 °C and 5% CO_2_.

### Lentiviral transfection

A stable *ZNF334* overexpressing cell line (ZNF334-OE) and its control cell line (ZNF334-NC) were constructed in the Huh7 cell line; A stable *ZNF334* knockdown cell line (sh-ZNF334) and its control cell line (sh-NC) were constructed in the SNU387 cell line. The *ZNF334* overexpression lentivirus is synthesized and packaged by Gene Pharma (Shanghai, China). Its overexpression vector is EF1a-CMV-GFP-T2A-puro, and the virus titer is 1.0E + 8. The *ZNF334* knockdown lentivirus was synthesized and packaged by Genechem (Shanghai, China). The shRNA vector was hU6-MCS-Ubiquitin-EGFP-IRES-puromycin, and the virus titer was 1.0E + 9. The interference fragment was 1#GAGGGCAAUUCUCAUUACATT; and 2#UGUAAUGAGAAUUGCCCUCTT. When the cell confluence is about 60%, virus solution and PolyBrene (5 μg/ml) were added to promote infection. For determination of the infection efficiency, fluorescence microscope was used after 72 h, and the cells were selected with puromycin.

### Mice in vivo limiting dilution assay and DEN-induced carcinogenesis mice model

Male mice (nude), 6–8 weeks old and male mice (C57BL/6), 1-week-old were purchased from Animal Research Center of Naval Medical University. For mice in vivo limiting dilution assay, the 48 nude mice were randomly divided into 8 groups, 6 in each group. Nude mice in each group were injected under the axilla of the right forelimb with the ZNF334-OE cells or its control cells (ZNF334-NC) at 5 × 10^6^, 1 × 10^6^, 5 × 10^5^, 1 × 10^5^ cells/group respectively. Subcutaneous tumors of the mice were collected after 2 months. The number and size of tumors were recorded. The website ELDA (http://bioinf.wehi.edu.au/software/elda/) was used to analyze the proportion of cancer stem cells in the mouse subcutaneous tumors and evaluate its sphere formation ability. For DEN-induced carcinogenesis mice model, the 30 C57BL/6 mice were divided into 2 groups, 15 in each group. The chemical HCC was induced by the combination of DEN (25 mg/kg i.p.) given at week 2 postpartum followed by weekly injections of CCl_4_ (0.5 mL/kg i.p., dissolved in olive oil). Mice were sacrificed at the indicated time for further studies. All animal experiments were approved by the Animal Care Committee of Naval Medical University, and the investigation complied with the Guide for the Care and Use of Laboratory Animals of the U.S. National Institutes of Health.

### MassArray methylation quantitative detection

MassARRAY methylation detection technology (Sequenom, USA) performed by CapitalBio Technology (Shanghai, China) was used to detect the methylation level of *ZNF334* promoter (human) in carcinoma and paracancerous tissues of 25 HCC patients and *Zfp334* promoter (mice) in treatment and control group. For mice *Zfp334* promoter, three primers (mouse-6, mouse-14, and mouse-16) were designed to cover all the CpGs. The primers were listed in Supplementary Table 2.

### Quantitative real-time PCR

Total RNA of the cells was extracted with the Trizol (Invitrogen), and the cDNA synthesis was completed with the TAKARA reverse transcription kit (TAKARA0360A). StepOne Plus(Applied Biosystem) was used for RT-qPCR experiments. The primers are listed in Supplementary Table 2.

### Western blot

Cells were lysed with RIPA Lysis Buffer (Beyotime, P0013C) to obtain total protein and then quantified by BCA method, and then 20 μg of each sample was subjected to SDS-PAGE electrophoresis and transferred to PVDF membrane for exposure. The antibodies used are as follows: Anti-ZNF334 (Abcam, #ab127712), Anti-GAPDH (Abcam, # ab8245), IRDye800CW goat anti-rabbit IgG (Licor, #926-3221), IRDye 680LT goat anti-mouse IgG (Licor, #926-68020).

### Dual-Luciferase reporter assay

The dual luciferase reporter plasmid psiCHECKTM-2 was synthesized by Gene Pharma (Shanghai, China). The promoter region of *ZNF334* is defined as the transcription start site to the upstream 2000 bp. Dual-Luciferase Reporter Assay System (Promega, E1910) for dual-luciferase reporter gene detection was used according to the user manual. The HepG2 cells that had been transfected with dual fluorescence and transcription factor plasmids or control plasmids were lysed with lysis buffer, and then firefly fluorescence and renilla fluorescence values were detected in a microplate reader, and statistical analysis was performed to compare the differences between the groups. The primer sequence of *ZNF334* promoter were listed in Supplementary Table 2.

### Chromatin immunoprecipitation assay

SimpleChIP^®^ Enzymatic Chromatin IP Kit (CST, Magnetic Beads, #9003) was used according to the user manual. Cells were cross-linked with 35% formaldehyde, immunoprecipitated, de-cross-linked, and DNA purified, and then subjected to RT-qPCR detection. The antibodies used are as follows: Anti-TP53 antibody (Active Motif, # AB_2793254). The primers were listed in Supplementary Table 2.

### Immunofluorescence staining

Cells were seeded on the slide of 6-well plates. When the cell confluence is about 60%, primary antibody (Abcam, #ab127712), secondary antibody (Licor, #926-3221) and DAPI were used according to the user manual to stain the cells. Confocal laser microscope was used for taking pictures.

### Cell proliferation assay (CCK8 and EdU)

Cells were seeded (2000/well) and maintained in 96-well plates. For determination of cell proliferation at indicated time points (0, 12, 24, 48, 60 and 72 h after adherence), a Cell-Counting Kit 8 (Beyotime, C0038) was used according to the user manual. Fluorescence (450 nm) was recorded in a microplate reader.

Cells were seeded on the slide of 6-well plates. When the cell confluence is about 50%, for determination of cell proliferation, a Cell-Light EdU Apollo643 In Vitro Kit (100 T) (RIBOBIO, C10310-2) was used according to the user manual. The slides were fixed, stained and observed under a microscope to obtain images.

### Cell apoptosis detection (TUNEL)

Cells were seeded on the slide of 6-well plates. When the cell confluence is about 50%, for determination of cell apoptosis, a TUNEL Apoptosis Detection Kit (Beyotime, C1086) was used according to the user manual. The slides were fixed, stained and observed under a microscope to obtain images.

### Flow cytometry

Annexin V: FITC Cell Apoptosis Detection Kit (BD, #556547) was used to determinate the cell apoptosis. Cells were collected, blocked with non-specific antigen, incubated with direct-labeled flow cytometry antibody, and then performed in BD LSRII.

### Plate clone formation assay

Cells were seeded (2000/well) and maintained in 6-well plates with FBS-free medium for 2 weeks and then fixed with formaldehyde, stained with crystal violet. The number of cell clusters in each well was counted and photographed.

### Sphere formation assay

Cells were seeded (2000/well) and maintained in low-attachment 6-well plates with FBS-free medium for 2 weeks. The number of spheroids and the size of cell clumps, were recorded under a microscope.

### Extra limiting dilution assay

Cells were resuspended and seeded (100, 200, 400, 800/well for 48, 24, 12, 6 wells respectively) in low-attachment 6-well plates with FBS-free medium for 2 weeks. The number of wells containing spheroids were counted and recorded under a microscope. The website ELDA (http://bioinf.wehi.edu.au/software/elda/) was used to analyze the proportion of cancer stem cells.

### Statistical analysis

All the data were expressed as mean ± SD for at least three independent experiments and were analyzed by two-sided Student’s *t* test, one-way ANOVA or two-way ANOVA. Non-parametric test was used when data does not meet the normal distribution and homogeneity of variance. The correlation between the methylation level and mRNA expression was tested by Pearson correlation coefficient. Log rank test and Cox regression model were used in the survival analysis. The survival curve was drawn using the Kaplan-Meier method. All statistical analysis was performed using GraphPad Prism 7.0 and relevant R packages. Probability (*P*) values ≤ 0.05 were considered to be statistically significant. *p < 0.05; **p < 0.01; ***p < 0.001; ****p < 0.0001.

## Supplementary information

supplementary information

aj-checklist.pdf

## Data Availability

The datasets used and analyzed during the current study are available from the corresponding author on reasonable request.

## Code availability

The code in this article can be obtained from https://cran.r-project.org and used by R studio software.
