# Supplementary material for: DNA hypermethylation modification promotes the development of hepatocellular carcinoma by depressing the tumor suppressor gene ZNF334
Source: Cell Death Dis. 2022 May 9;13(5):446. doi: 10.1038/s41419-022-04895-6 (PMC9085879; doi:10.1038/s41419-022-04895-6)

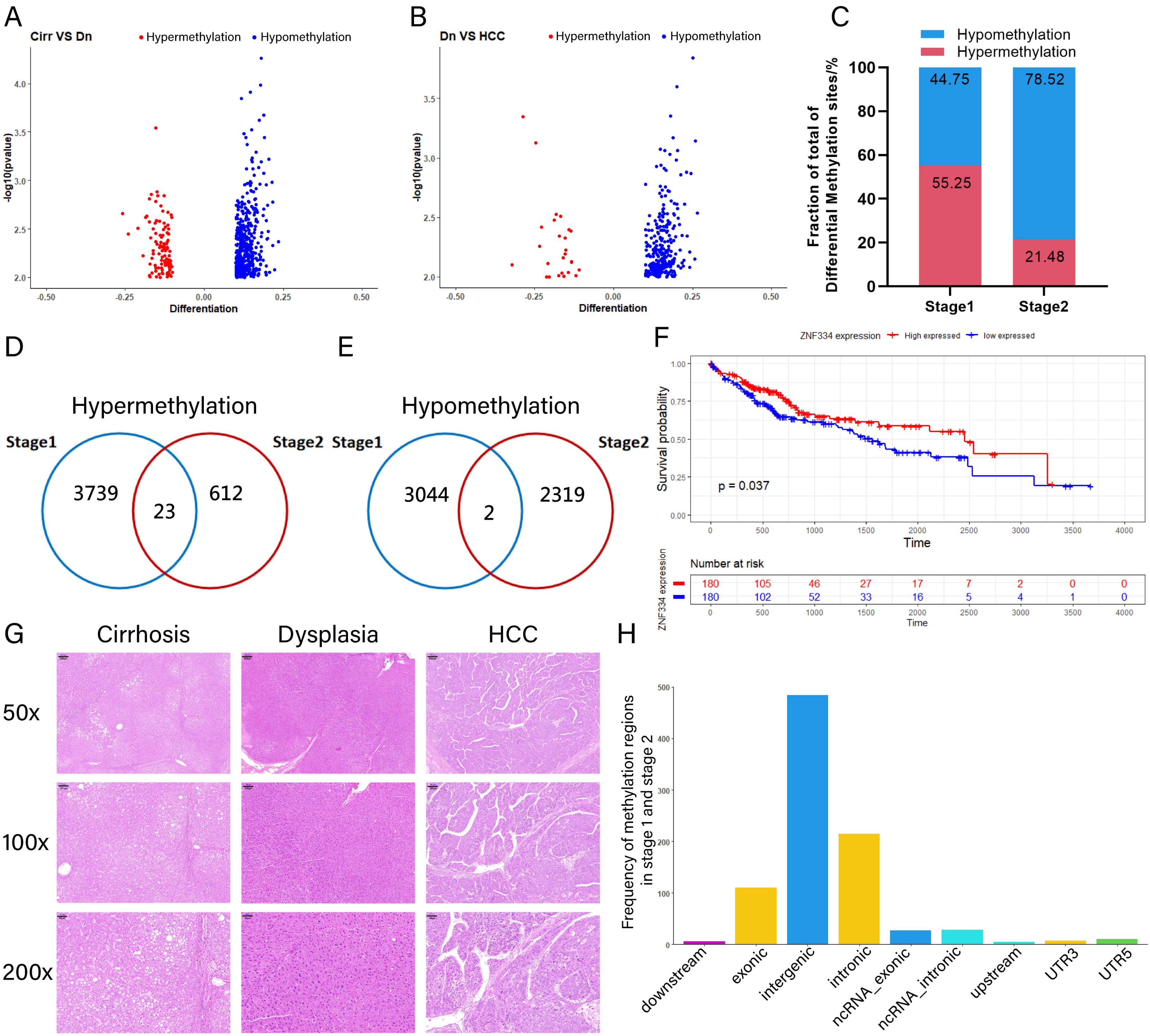

A
Cirr VS Dn
Hypermethylation
Hypomethylation
-log10(pvalue)
Differentiation
B
Dn VS HCC
Hypermethylation
Hypomethylation
-log10(pvalue)
Differentiation
C
Hypomethylation
Hypermethylation
Fraction of total of Differential Methylation sites/%
44.75
78.52
55.25
21.48
Stage1
Stage2
D
Hypermethylation
Stage1
Stage2
3739
23
612
E
Hypomethylation
Stage1
Stage2
3044
2
2319
F
ZNF334 expression
High expressed
low expressed
Survival probability
p = 0.037
Time
Number at risk
ZNF334 expression
180 105 46 27 17 7 2 0 0
180 102 52 33 16 5 4 1 0
Time
G
Cirrhosis
Dysplasia
HCC
50x
100x
200x
H
Frequency of methylation regions in stage 1 and stage 2
downstream
exonic
intergenic
intronic
ncRNA_exonic
ncRNA_intronic
upstream
UTR3
UTR5

A

B

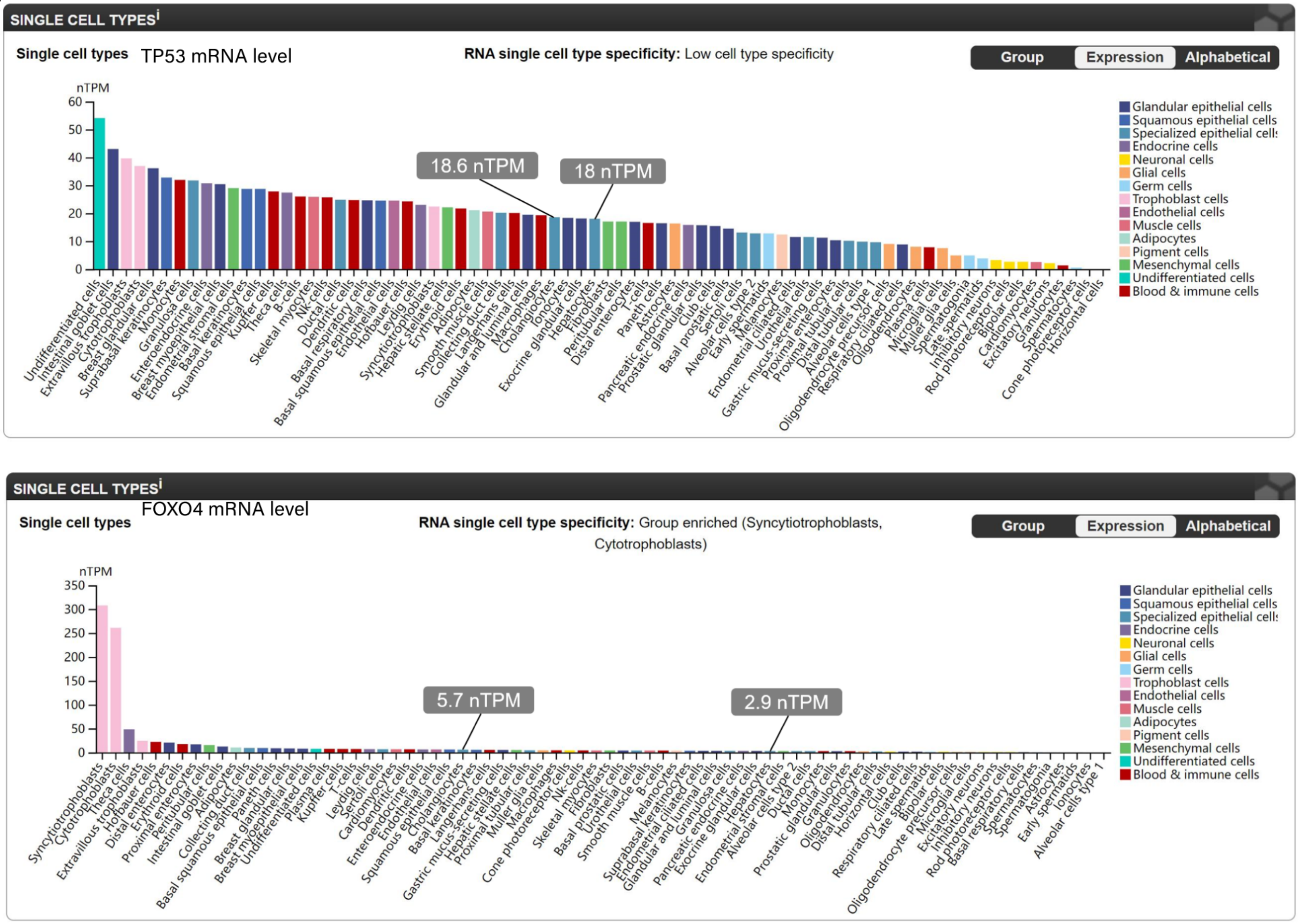
SINGLE CELL TYPES
Single cell types
TP53 mRNA level
RNA single cell type specificity: Low cell type specificity
Group
Expression
Alphabetical
nTPM
60
50
40
30
20
10
0
18.6 nTPM
18 nTPM
Glandular epithelial cells
Squamous epithelial cells
Specialized epithelial cells
Endocrine cells
Neuronal cells
Glial cells
Germ cells
Trophoblast cells
Endothelial cells
Muscle cells
Adipocytes
Pigment cells
Mesenchymal cells
Undifferentiated cells
Blood & immune cells
SINGLE CELL TYPES
FOXO4 mRNA level
Single cell types
RNA single cell type specificity: Group enriched (Syncytiotrophoblasts, Cytotrophoblasts)
Group
Expression
Alphabetical
nTPM
350
300
250
200
150
100
50
0
5.7 nTPM
2.9 nTPM
Glandular epithelial cells
Squamous epithelial cells
Specialized epithelial cells
Endocrine cells
Neuronal cells
Glial cells
Germ cells
Trophoblast cells
Endothelial cells
Muscle cells
Adipocytes
Pigment cells
Mesenchymal cells
Undifferentiated cells
Blood & immune cells

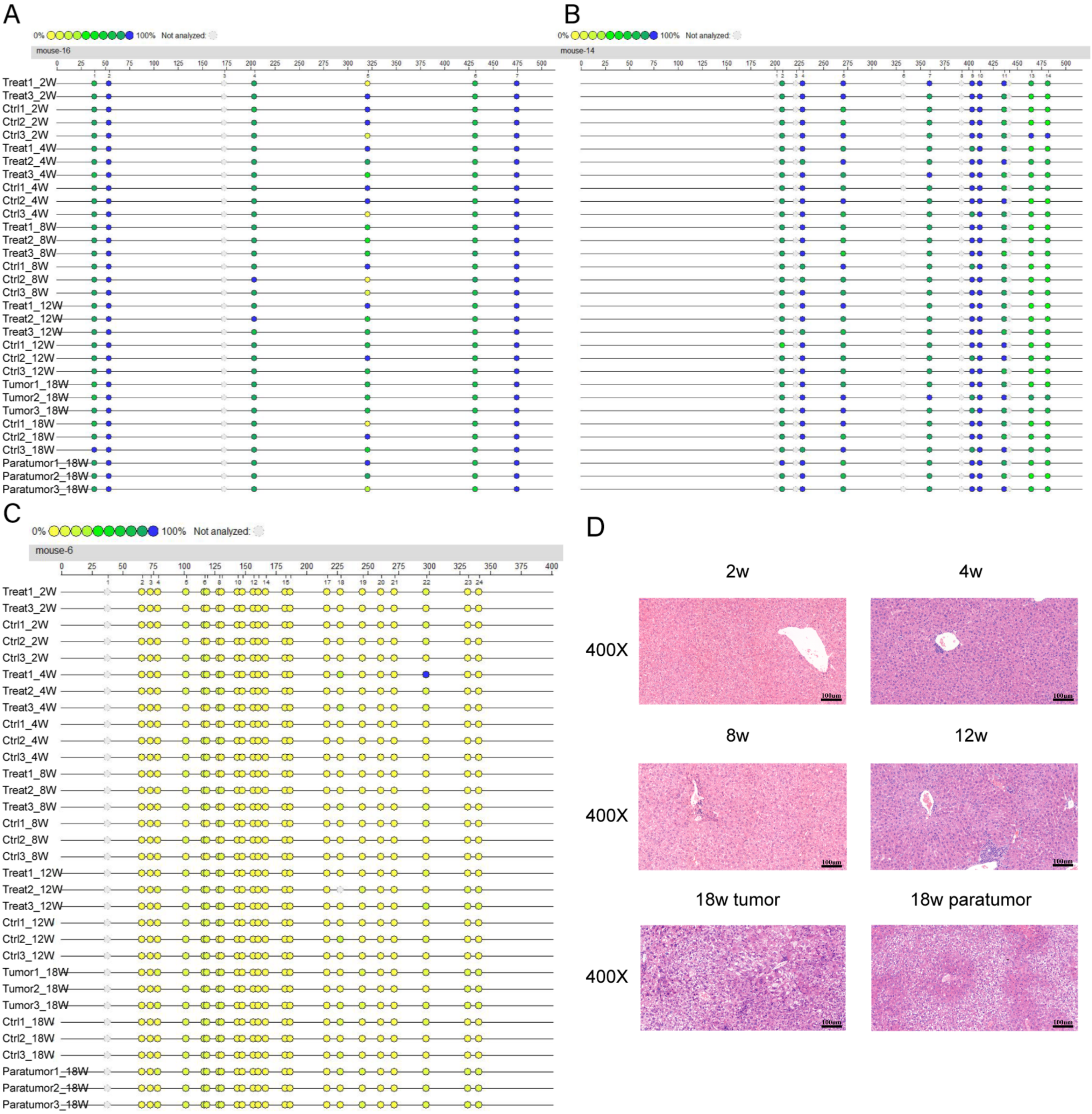
A
0%
100%
Not analyzed:
mouse-16
B
mouse-14
C
mouse-6
Treat1_2W
Treat3_2W
Ctrl1_2W
Ctrl2_2W
Ctrl3_2W
Treat1_4W
Treat2_4W
Treat3_4W
Ctrl1_4W
Ctrl2_4W
Ctrl3_4W
Treat1_8W
Treat2_8W
Treat3_8W
Ctrl1_8W
Ctrl2_8W
Ctrl3_8W
Treat1_12W
Treat2_12W
Treat3_12W
Ctrl1_12W
Ctrl2_12W
Ctrl3_12W
Tumor1_18W
Tumor2_18W
Tumor3_18W
Ctrl1_18W
Ctrl2_18W
Ctrl3_18W
Paratumor1_18W
Paratumor2_18W
Paratumor3_18W
D
2w
4w
8w
12w
18w tumor
18w paratumor
400X
100um

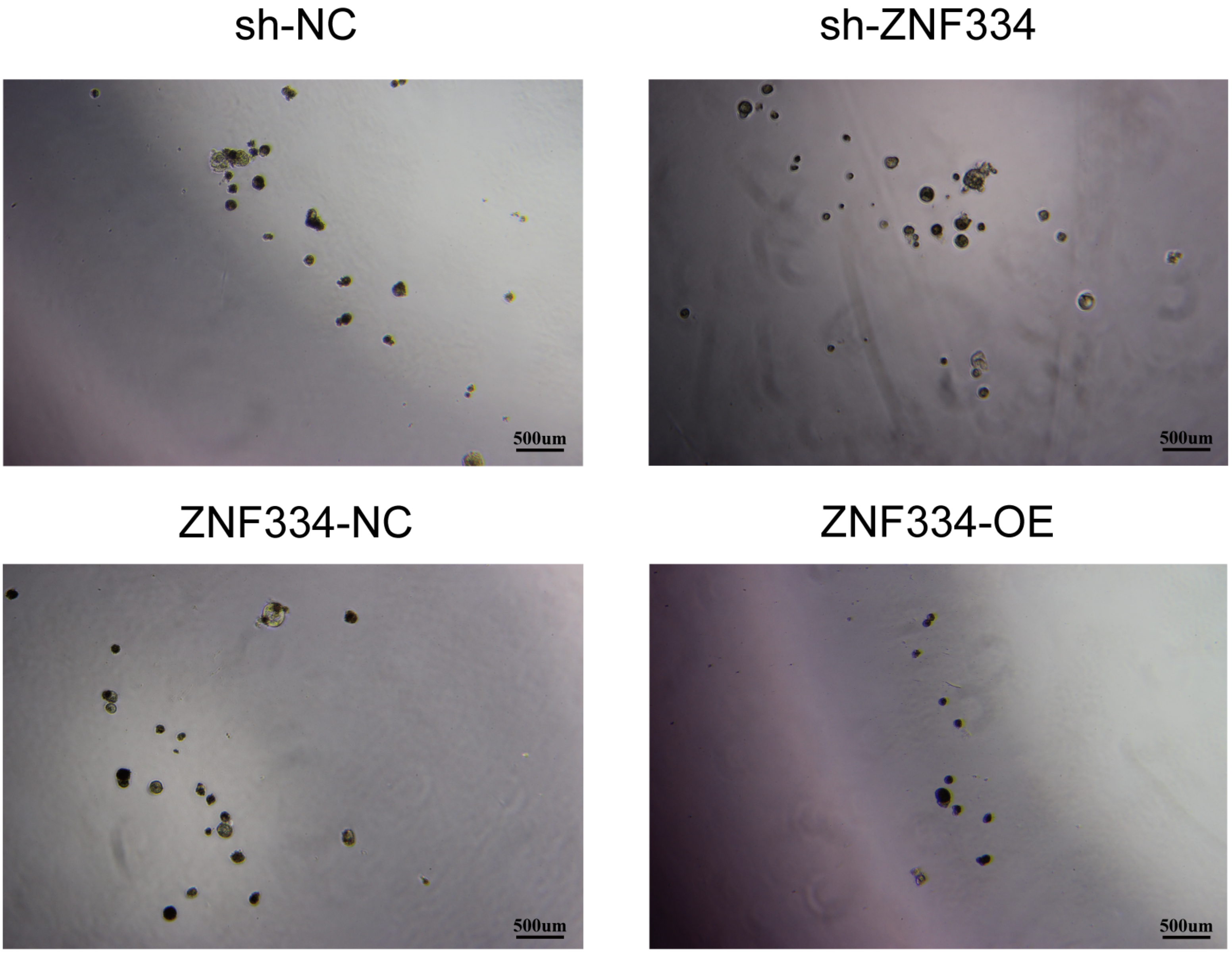
sh-NC
sh-ZNF334
500um
500um
ZNF334-NC
ZNF334-OE
500um
500um

Figure S1. DNA methylation analysis reveals differentially methylated sites and regions in different stages of different types of tissues. A. Volcano plot of differentiation of differentially methylated regions from Cirrhosis to dysplastic nodules. Red dots are hypermethylated regions, blue dots are hypomethylated regions. B. Volcano plot of differentiation of differentially methylated regions from dysplastic nodules to HCC. Red dots are hypermethylated regions, blue dots are hypomethylated regions. C. The abundance of differentially methylated sites in the comparison of the first and second stages. D. Venn diagram of Hypermethylated sites in the first and second stages. E. Venn diagram of hypomethylated sites in the first and second stages. F. Kaplan–Meier curves of differentially expressed and not differentially expressed groups for ZNF334. LIHC cases downloaded from TCGA were divided into two groups based on their expression level: the top half of 156 cases were differentially expressed and the bottom half of 157 cases are not differentially expressed. G. The typical HE staining picture of cirrhosis tissue, dysplastic nodules and HCC tissues at different picture magnification. H. Number of different types of differentially methylated regions in the first and second stages.

Figure S2. A summary of single cell RNA(nTPM) from all single cell types exhibited in the Human Protein Atlas database. A. The expression level of TP53 in different type of cells. B. The expression level of FOXO4 in different type of cells

Figure S3. Methylation degree of Zfp334 promoter at different methylation sites

and HE staining of DEN-induced carcinogenesis mice model. A. Methylation degree of Zfp334 promoter at different methylation sites using mouse-16 primer. B. Methylation degree of Zfp334 promoter at different methylation sites using mouse-14 primer. C. Methylation degree of Zfp334 promoter at different methylation sites using mouse-4 primer. D. The typical HE staining pictures of DEN-induced carcinogenesis mice model at 2w, 4w, 8w, 12w, 18w after first CCl_4 injection and the picture magnification was 400×.

Figure S4. Representative images of ZNF334-OE, ZNF334-NC, sh-ZNF334, and sh-NC by sphere formation assay at a magnification of 40×.

Supplementary table 1. Enriched functions of hypermethylated genes by AutoCompare_ZE software based on Reactome pathway database.

Pathway	FE *p* value	ZE *p* value	Number of common genes	Common Genes
**REACTOME_SIGNALING_BY_WNT**	**6.55e-05**	**2.34e-05**	**15/329**	**HECW1,ZRANB1,CSNK2A2,PPP2R5C,TNRC6A,TNRC6B,RNF43,PDE6A,APC,TLE3,WNT3A,FZD1,LRP5,YWHAZ,PPP3R1**
REACTOME_SIGNALING_BY_WNT_IN_CANCER	1.07e-04	1.78e-04	5/34	PPP2R5C,RNF43,APC,WNT3A,LRP5
REACTOME_SIGNALING_BY_RNF43_MUTANTS	1.49e-04	2.76e-04	3/8	,RNF43,WNT3A,LRP5
REACTOME_NEGATIVE_REGULATION_OF_ACTIVITY_OF_TFAP2_AP_2_FAMILY_TRANSCRIPTION_FACTORS	8.35e-03	8.41e-03	2/10	TFAP2D,TFAP2E
REACTOME_INTRINSIC_PATHWAY_FOR_APOPTOSIS	7.57e-03	1.32e-02	4/55	YWHAQ,CDKN2A,YWHAZ,PPP3R1
**REACTOME_TP53_REGULATES_METABOLIC_GENES**	**7.82e-03**	**1.61e-02**	**5/87**	**TNRC6A,TNRC6B,GLS,YWHAQ,YWHAZ**
REACTOME_ONCOGENE_INDUCED_SENESCENCE	1.31e-02	1.62e-02	3/35	TNRC6A,TNRC6B,CDKN2A
REACTOME_NOTCH4_ACTIVATION_AND_TRANSMISSION_OF_SIGNAL_TO_THE_NUCLEUS	1.01e-02	1.70e-02	2/11	JAG1,YWHAZ
REACTOME_REGULATION_OF_LOCALIZATION_OF_FOXO_TRANSCRIPTION_FACTORS	1.20e-02	1.71e-02	2/12	YWHAQ,YWHAZ
**REACTOME_CHK1_CHK2_CDS1_MEDIATED_INACTIVATION_OF_CY**	**1.41e-02**	**2.07e-02**	**2/13**	**YWHAQ,YWHAZ**

**CLIN_B_CDK1_COMPLEX**				
REACTOME_SIGNALING_BY_CTNNB1_PHOSPHO_SITE_MUTANTS	1.86e-02	2.24e-02	2/15	PPP2R5C,APC
REACTOME_REGULATION_OF_RUNX1_EXPRESSION_AND_ACTIVITY	2.36e-02	2.99e-02	2/17	TNRC6A,TNRC6B
REACTOME_RECEPTOR_TYPE_TYROSINE_PROTEIN_PHOSPHATASES	3.21e-02	3.11e-02	2/20	PTPRD,PPFIA3
REACTOME_MECP2_REGULATES_NEURONAL_RECEPTORS_AND_CHANNELS	2.64e-02	3.57e-02	2/18	OPRK1,FKBP5
REACTOME_COLLAGEN_CHAIN_TRIMERIZATION	2.42e-02	3.85e-02	3/44	COL17A1,COL26A1,COL27A1
REACTOME_NR1H2_AND_NR1H3_MEDIATED_SIGNALING	2.88e-02	3.95e-02	3/47	TNRC6A,TNRC6B,EEPD1
REACTOME_CELL_CELL_COMMUNICATION	3.75e-02	4.34e-02	5/130	LAMC2,COL17A1,CDH2,JUP,SIRPA
REACTOME_CELL_JUNCTION_ORGANIZATION	4.16e-02	4.40e-02	4/92	LAMC2,COL17A1,CDH2,JUP
REACTOME_RAS_ACTIVATION_UPON_CA2_INFLUX_THROUGH_NMDA_RECEPTOR	3.21e-02	4.64e-02	2/20	RASGRF2,DLG2
REACTOME_NOTCH1_INTRACELLULAR_DOMAIN_REGULATES_TRANSCRIPTION	3.04e-02	4.80e-02	3/48	HDAC7,TLE3,MAML3

Supplementary table 2. Primers used in this article

Primer names	Sequences
MassArray methylation	
ZNF334-5#-F	aggaagagagGGTTAGGAGTTTAATTTTGTTTTGGT

ZNF334-5#-R	cagtaatacgactcactatagggagaaggctAACTCCTCAAAAATCCCTCAAAATA
Zfp334-6#-F	aggaagagagGGGTTTTTTGGGAGTTATAAAAGAA
Zfp334-6#-R	cagtaatacgactcactatagggagaaggctAATACAAAAACCTTTTTCTCAACCA
Zfp334-14#-F	aggaagagagTTGTTGGTTTGTTTTTTTAGGTTTT
Zfp334-14#-R	cagtaatacgactcactatagggagaaggctAATCTACTTCCCTTACTTTCCCTAA
Zfp334-16#-F	aggaagagagGAAGGTATTGTTAGATGTGTTGGAG
Zfp334-16#-R	cagtaatacgactcactatagggagaaggctAAAAAAAACAACAACAAACACCATT

Realtime quantitative PCR

18S-F	GGAGAGGGAGCCTGAGAAACG
18S-R	TTACAGGGCCTCGAAAGAGTCC
ACTB-F	CCACCATGTACCCTGGCATTG
ACTB-R	TCATCTTGTTTTCTGCGCAAGTTA
CD133-F	AGTCGGAAACTGGCAGATAGC
CD133-R	GGTAGTGTTGTACTGGGCCAAT
CD24-F	CTCCTACCCACGCAGATTTATTC
CD24-R	AGAGTGAGACCACGAAGAGAC
CD44-F	CTGCCGCTTTGCAGGTGTA
CD44-R	CATTGTGGGCAAGGTGCTATT
CD90-F	ATCGCTCTCCTGCTAACAGTC
CD90-R	CTCGTACTGGATGGGTGAACT
EpCAM-F	AATCGTCAATGCCAGTGTACTT
EpCAM-R	TCTCATCGCAGTCAGGATCATAA

GAPDH-F	AGCGAGCATCCCCCAAAGTT
GAPDH-R	GGGCACGAAGGCTCATCATT
ZNF334-F	AGGGGAGACAGACTGAAAGGA
ZNF334-R	GTGAGGCTTGTCTTCACACG

Truncation primers for ZNF334 promoters

promoter-2.0k-F	CATGGCTCGACAGATCTTGTTCCCAATTACAAAAG
promoter-2.0k-R	TTGGAAGCCATGGTGGCTAGCTGGCGAACCGGAAGGGCG
promoter-1.5k-F	CATGGCTCGACAGATCTTGTTCCCAATTACAAAAG
promoter-1.5k-R	TTGGAAGCCATGGTGGCTAGCGGAACTCATATAGGTCTAG
promoter-1.0k-F	CATGGCTCGACAGATCTTGTTCCCAATTACAAAAG
promoter-1.0k-R	TTGGAAGCCATGGTGGCTAGCGAATAAATAGAA CTTTA
promoter-0.5k-F	CATGGCTCGACAGATCTTGTTCCCAATTACAAAAG
promoter-0.5k-R	TTGGAAGCCATGGTGGCTAGCAGTTCCTGGATGACTTCAG

Chromatin immunoprecipitation assay

ZNF-P53-1-F	TCTCTTTTTCCATGTGGTCTC
ZNF-P53-1-R	TTTGCCCTTTGAAGAGTCCTT
ZNF-P53-2-F	AAGTCAACGTGGGAGGGAAA
ZNF-P53-2-R	ATTCCAGAAAAGAACAATTAG
ZNF-P53-3-F	GTACGTTTACTGCACCTTCCC
ZNF-P53-3-R	ACAGGCCTGTGTGGCATGTCA
ZNF-P53-4-F	GTTACCATTGCATATGGTATTC
ZNF-P53-4-R	TCTAGACAATATAGTCTACTAC

ZNF-P53-5-F	AATAACAAATTACTAGACCTATA
ZNF-P53-5-R	TCAGAATATCTCTGAAGAAA
ZNF-P53-6-F	TTCTGGGGACTGTGGTCCGGAA
ZNF-P53-6-R	GTCCAGGTAAAAAACAGGA

Supplementary table 3. Correlations between ZNF334 expression and clinical characteristics in HCC patients (n = 213)

Characteristics	Total *N=213*	ZNF334 expression high expression *N=107*	ZNF334 expression low expression *N=106*	*p*
Gender:				0.177
Male	183 (85.9%)	88 (82.2%)	95 (89.6%)	
Female	30 (14.1%)	19 (17.8%)	11 (10.4%)	
Age(years):				0.587
<60	146 (68.5%)	71 (66.4%)	75 (70.8%)	
>=60	67 (31.5%)	36 (33.6%)	31 (29.2%)	
Pathologic grade:				0.454
II stage	31 (14.6%)	18 (16.8%)	13 (12.3%)	
III&IV stage	182 (85.4%)	89 (83.2%)	93 (87.7%)	
Tumor diameter(cm):				0.303
<5	103 (48.4%)	56 (52.3%)	47 (44.3%)	
>=5	110 (51.6%)	51 (47.7%)	59 (55.7%)	

Characteristics	Total<br>*N=213*	ZNF334 expression		*p*
		**high expression**<br>*N=107*	**low expression**<br>*N=106*	
Number of tumors:				**0.011**
Multiple	55 (25.8%)	19 (17.8%)	36 (34.0%)	
Single	158 (74.2%)	88 (82.2%)	70 (66.0%)	
Tumor capsule:				**<0.001**
Negative	108 (50.7%)	40 (37.4%)	68 (64.2%)	
Positive	105 (49.3%)	67 (62.6%)	38 (35.8%)	
MVI:				0.724
Negative	119 (55.9%)	58 (54.2%)	61 (57.5%)	
Positive	94 (44.1%)	49 (45.8%)	45 (42.5%)	
Portal vein tumor thrombus:				**0.005**
Negative	75 (35.2%)	48 (44.9%)	27 (25.5%)	
Positive	138 (64.8%)	59 (55.1%)	79 (74.5%)	
CEA(ng/ml):				0.621
<10	209 (98.1%)	104 (97.2%)	105 (99.1%)	
>=10	4 (1.88%)	3 (2.80%)	1 (0.94%)	
Albumin(g/L):				0.371
<35	42 (19.7%)	18 (16.8%)	24 (22.6%)	
>=35	171 (80.3%)	89 (83.2%)	82 (77.4%)	
ALT(U/L):				**0.014**

Characteristics	Total<br>*N=213*	ZNF334 expression		*p*
		high expression<br>*N=107*	low expression<br>*N=106*	
<=40	131 (61.5%)	75 (70.1%)	56 (52.8%)	
>40	82 (38.5%)	32 (29.9%)	50 (47.2%)	
AST(U/L):				**0.001**
<=40	149 (70.0%)	86 (80.4%)	63 (59.4%)	
>40	64 (30.0%)	21 (19.6%)	43 (40.6%)	
PT:				0.237
Normal	154 (72.3%)	73 (68.2%)	81 (76.4%)	
Abnormal	59 (27.7%)	34 (31.8%)	25 (23.6%)	
BCLC stage:				1.000
A&B stage	138 (64.8%)	69 (64.5%)	69 (65.1%)	
C stage	75 (35.2%)	38 (35.5%)	37 (34.9%)	
HBsAg:				0.220
Negative	29 (13.6%)	11 (10.3%)	18 (17.0%)	
Positive	184 (86.4%)	96 (89.7%)	88 (83.0%)	
Smoking:				1.000
Negative	98 (46.0%)	49 (45.8%)	49 (46.2%)	
Positive	115 (54.0%)	58 (54.2%)	57 (53.8%)	
Drinking:				0.225
Negative	131 (61.5%)	61 (57.0%)	70 (66.0%)	

Characteristics	Total *N=213*	ZNF334 expression		*p*
		high expression *N=107*	low expression *N=106*	
Positive	82 (38.5%)	46 (43.0%)	36 (34.0%)	
Cirrhosis:				0.467
Negative	20 (9.39%)	8 (7.48%)	12 (11.3%)	
Positive	193 (90.6%)	99 (92.5%)	94 (88.7%)	
AFP(ng/mL):				**0.035**
<400	146 (68.5%)	81 (75.7%)	65 (61.3%)	
>=400	67 (31.5%)	26 (24.3%)	41 (38.7%)	
CA19-9(U/mL):				0.190
<40	87 (79.8%)	31 (88.6%)	56 (75.7%)	
>=40	22 (20.2%)	4 (11.4%)	18 (24.3%)	
CRP(mg/L):				0.212
<10	49 (39.2%)	12 (30.0%)	37 (43.5%)	
>=10	76 (60.8%)	28 (70.0%)	48 (56.5%)	
TB(μmol/L):				0.305
<17.1	111 (52.1%)	60 (56.1%)	51 (48.1%)	
>=17.1	102 (47.9%)	47 (43.9%)	55 (51.9%)	

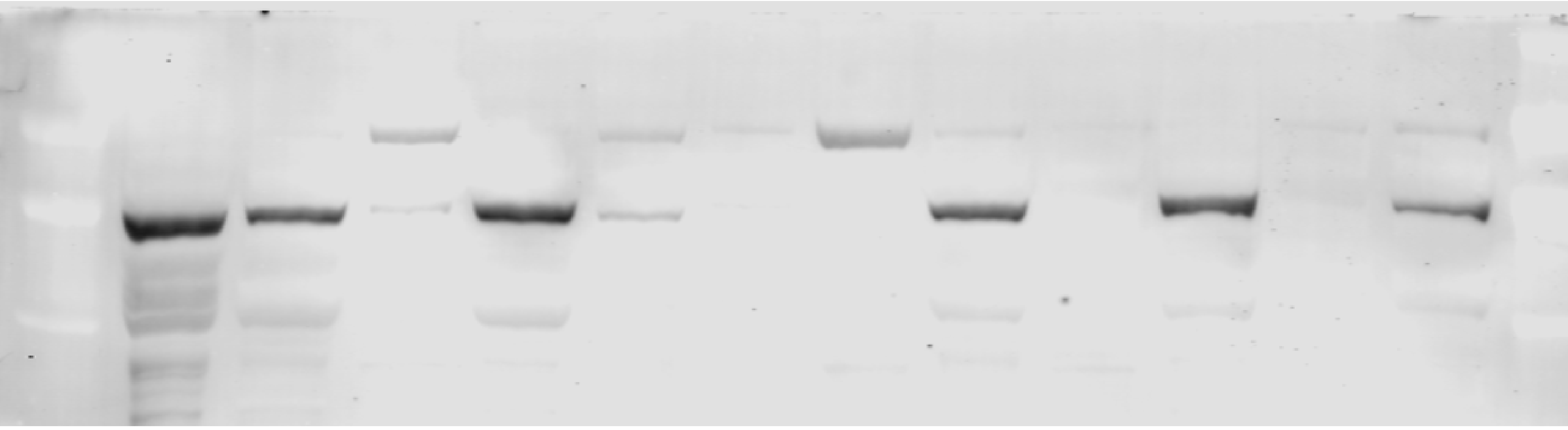

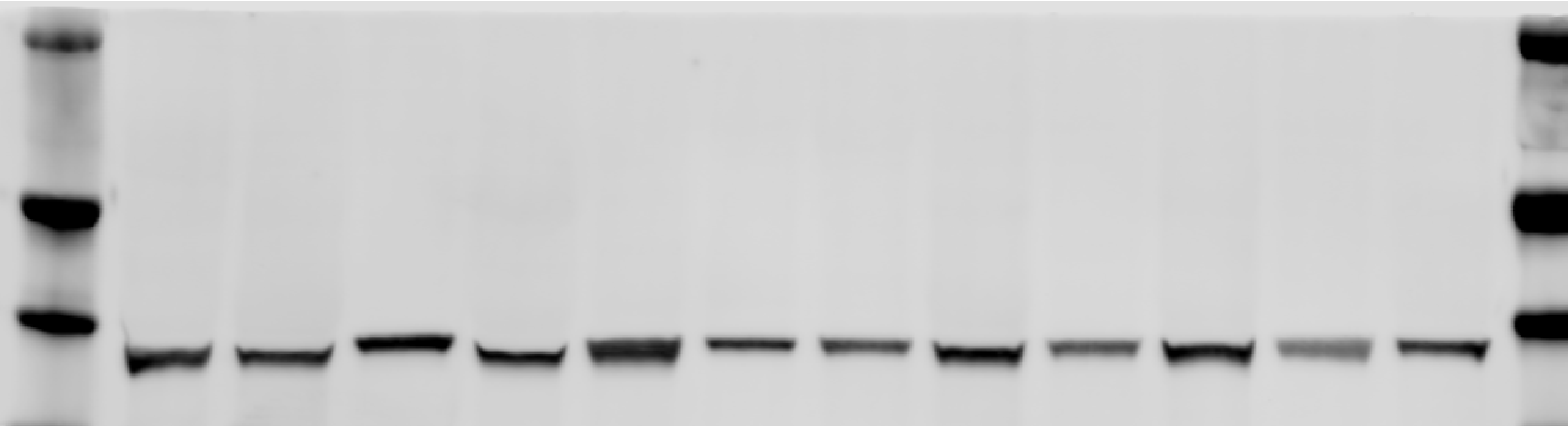

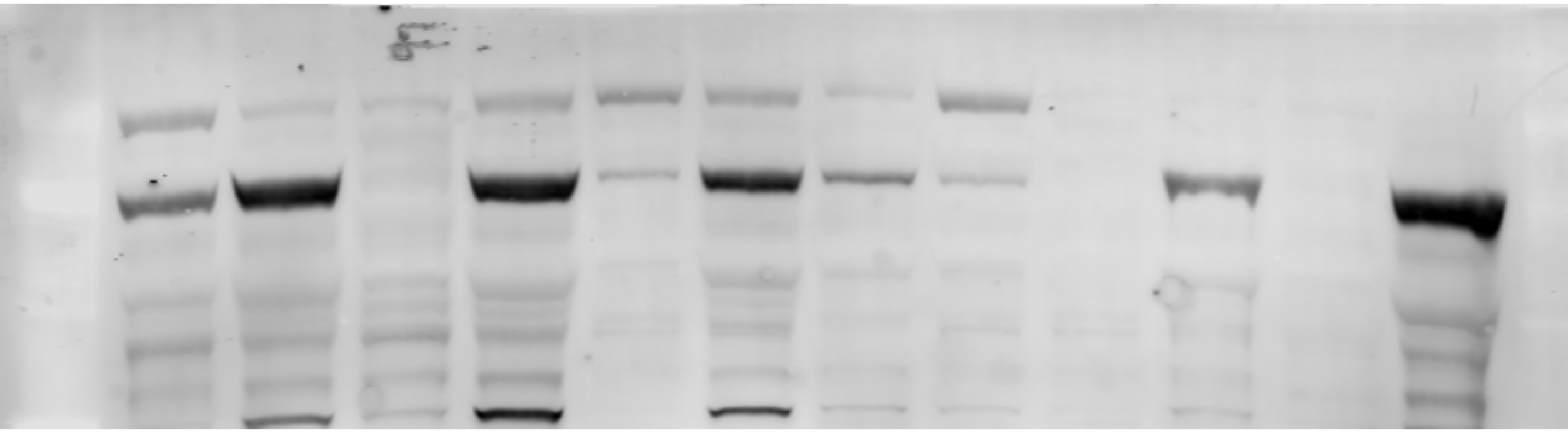

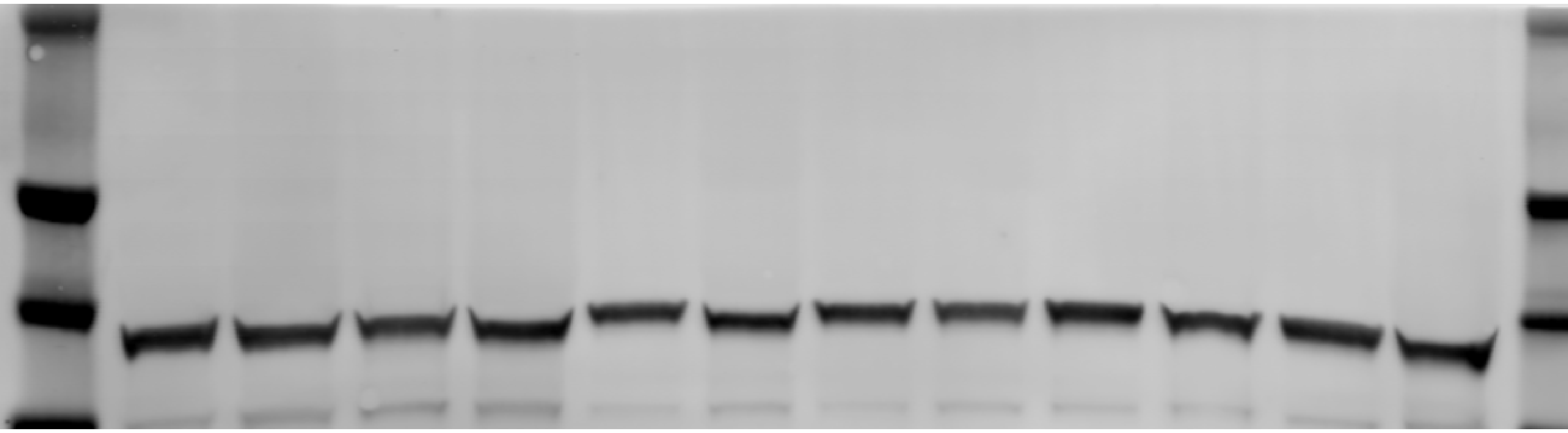

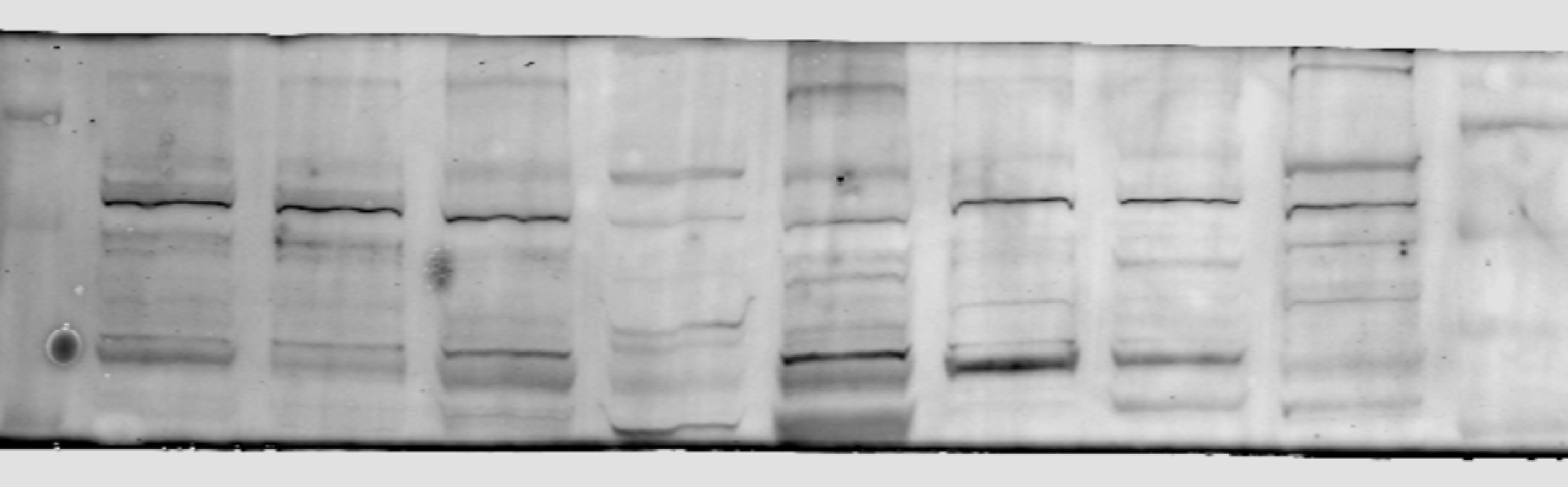

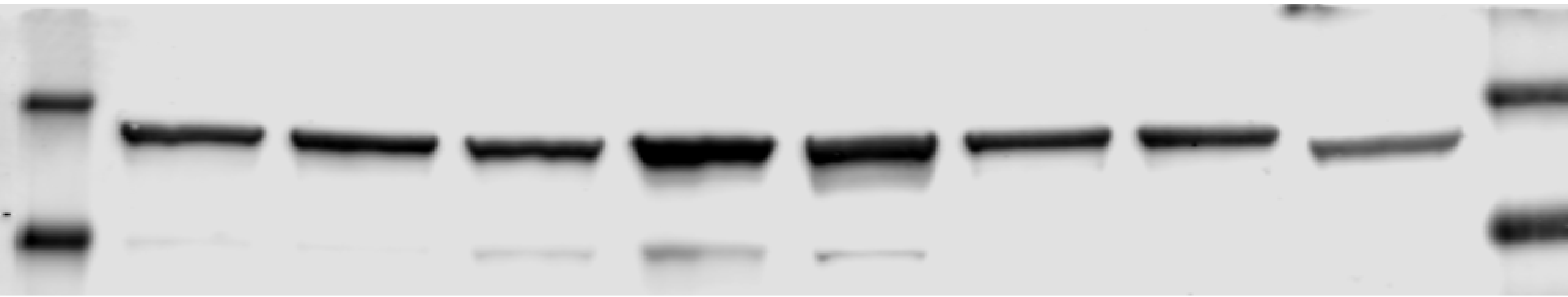

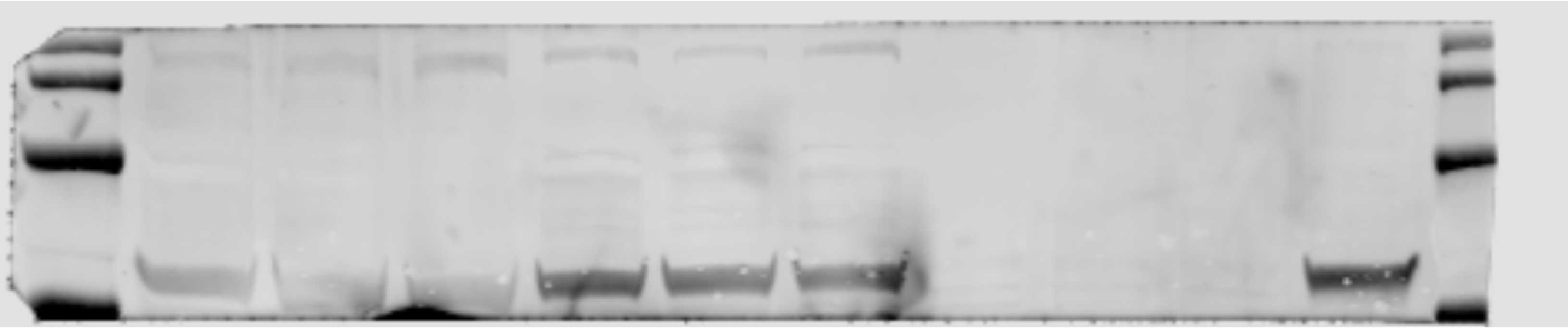

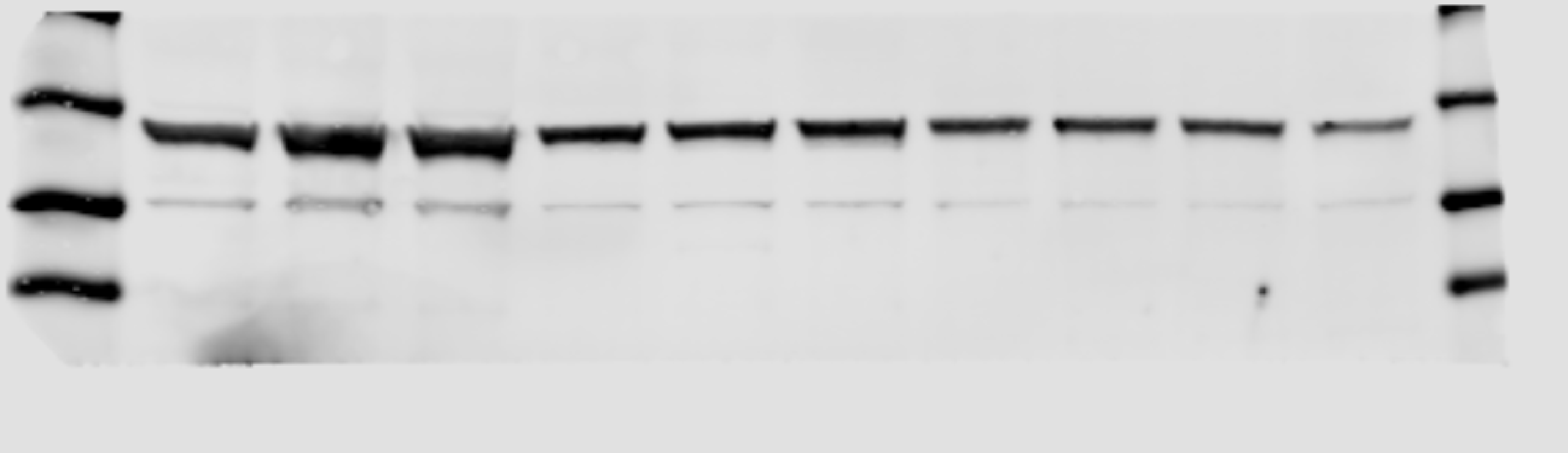

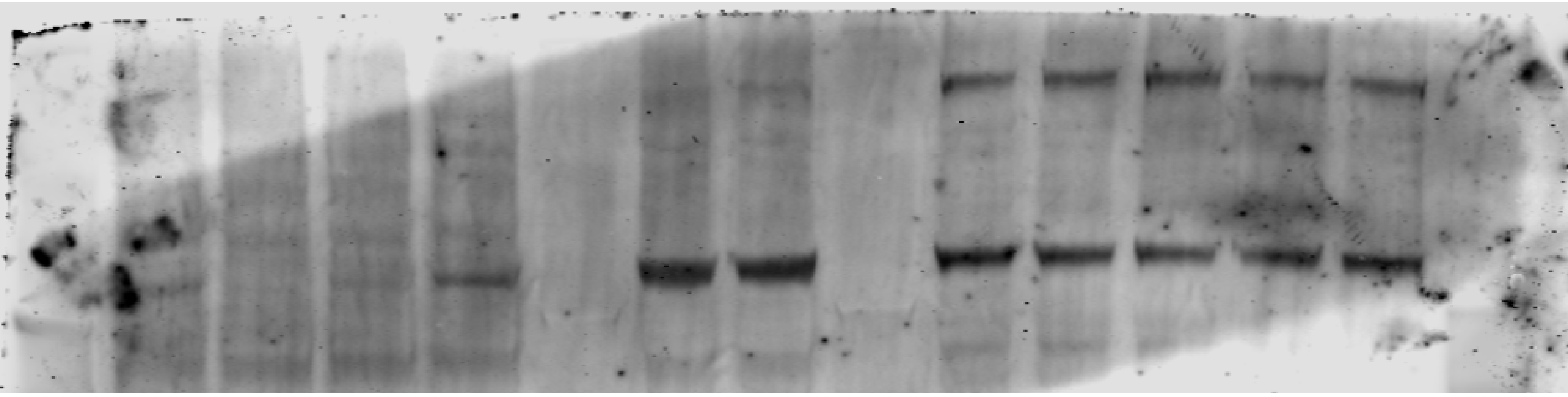

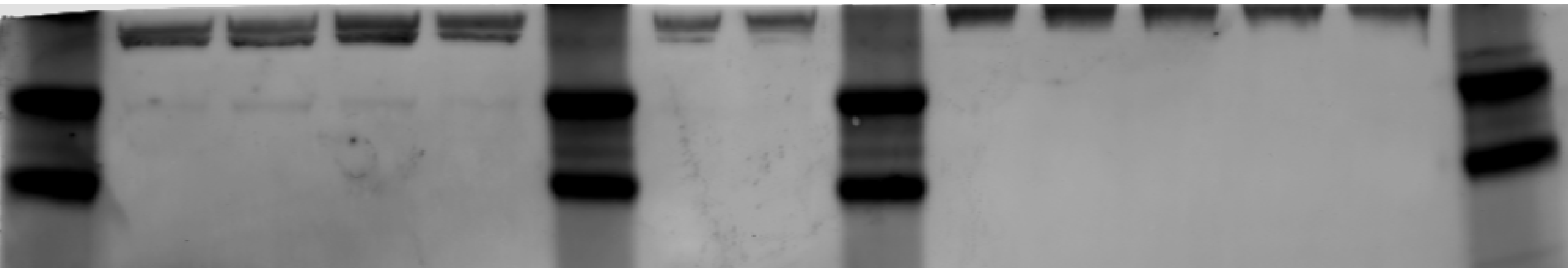

Supplement: Supplementary file 1 — supplementary information [file 41419_2022_4895_MOESM1_ESM.pdf]
